# Curcumin analogues as selective fluorescence imaging probes for brown adipose tissue and monitoring browning

**DOI:** 10.1038/srep13116

**Published:** 2015-08-13

**Authors:** Xueli Zhang, Yanli Tian, Hongbin Zhang, Amol Kavishwar, Matthew Lynes, Anna-Liisa Brownell, Hongbin Sun, Yu-Hua Tseng, Anna Moore, Chongzhao Ran

**Affiliations:** 1 Molecular Imaging Laboratory, Athinoula A. Martinos Center for Biomedical Imaging, Massachusetts General Hospital/Harvard Medical School, Boston, MA; 2 School of Pharmacy, China Pharmaceutical University, Nanjing 210009, China; 3 Department of pharmacy, ZhongDa Hospital, Southeast University, Nanjing 210009, China; 4 Department of Parasitology, Zhongshan School of Medicine, Sun Yat-Sen University, Guangzhou, China; 5 Joslin Diabetes Center, Harvard Medical School, and Harvard Stem Cell Institute, Boston, MA 02215.

## Abstract

Manipulation of brown adipose tissue (BAT) and browning of white adipose tissue (WAT) can be promising new approaches to counter metabolic disorder diseases in humans. Imaging probes that could consistently monitor BAT mass and browning of WAT are highly desirable. In the course of our imaging probe screening, we found that BAT could be imaged with curcumin analogues in mice. However, the poor BAT selectivity over WAT and short emissions of the lead probes promoted further lead optimization. Limited uptake mechanism studies suggested that CD36/FAT (fatty acid transporter) probably contributed to the facilitated uptake of the probes. By increasing the stereo-hindrance of the lead compound, we designed CRANAD-29 to extend the emission and increase the facilitated uptake, thus increasing its BAT selectivity. Our data demonstrated that CRANAD-29 had significantly improved selectivity for BAT over WAT, and could be used for imaging BAT mass change in a streptozotocin-induced diabetic mouse model, as well as for monitoring BAT activation under cold exposure. In addition, CRANAD-29 could be used for monitoring the browning of subcutaneous WAT (sWAT) induced by β3-adrenoceptor agonist CL-316, 243.

Brown adipose tissue (BAT), a specialized tissue involved in thermogenesis in mammals, is very important for maintaining energy balance in the whole body through dissipating large amounts of chemical/food energy as heat^1^,^2^,^3^. The most distinctive features of BAT include a large number of mitochondria, abundant uncoupling protein-1 (UCP-1), and numerous small oil droplets in a single cell, as well as highly vascularized vessel structures^4^,^5^,^6^,^7^,^8^. These characteristics strongly indicate that BAT plays an important role in metabolism and energy expenditure.

In humans, BAT is more abundant in embryonic and early postnatal stages, but is drastically reduced or was considered to have no physiological relevance in adult humans. However, its importance has recently “re-emerged” in new studies. Positron Emission Tomography (PET) imaging with ^18^F-FDG has shown that BAT is still present in adults in the upper chest, neck, and in other locations^4^,^5^,^6^,^9^,^10^,^11^,^12^,^13^,^14^,^15^. Cypess *et al*. imaged and analyzed 3,640 patients and showed that BMI (body mass index) inversely correlated with the amount of brown adipose tissue, suggesting that BAT is an important target in obesity and diabetes^4^. Other studies also demonstrated that both BMI and body fat percentage had a significant negative correlation with BAT, whereas resting metabolic rate correlated positively with BAT^9^,^16^. Therefore, manipulating BAT mass, which includes browning of WAT, BAT transplantation, and BAT mass promotion by drug treatment^6^,^17^,^18^,^19^,^20^,^21^,^22^,^23^,^24^, is a very attractive approach for obesity and diabetes therapies.

“Browning” is a process of inducing “brown fat-like” changes in white adipose tissue^15^,^19^,^20^,^22^,^23^,^25^,^26^. The appearances of multilocular and UCP1-positive fat cells within WAT are the characteristic changes during browning. Recent reports indicated that physical exercise could lead to browning of WAT in animal studies^27^,^28^. Moreover, several studies have shown that the β3-adrenoceptor agonist (CL316, 243), PPAR-gamma ligand (rosiglitazone), miRNAs, and genetic manipulation of PRDM16, BMP7, and PGC-1α could induce a “browning” process^19^,^20^,^21^,^26^,^28^,^29^,^30^,^31^. These results suggest the potential benefits of browning for obesity treatment. In addition, there are indications that BAT plays significant roles in ageing^16^,^32^,^33^, infection^34^, inflammation^35^, cardiovascular diseases, cancer, neurodegenerative diseases, and other disorders^6^,^32^,^36^,^37^,^38^.

Currently, the most widely used method for BAT imaging is PET imaging with ^18^F-FDG. However, BAT imaging with ^18^F-FDG requires pretreatment activation (such as cold stress or norepinephrine treatment), and most likely reflects the degree of activation, but not the amount of BAT mass^4^,^5^,^37^,^39^,^40^,^41^. A PET imaging probe for BAT thermogenesis has been reported^42^. MRI and CT have also been used for imaging BAT^37^,^43^,^44^,^45^, CEST MRI and MRS have been applied for measuring the white and brown tissue fraction in mice^46^, and hyperpolarized Xenon MRI also can be used to visualize BAT^47^. Compared to PET and MRI imaging, NIR Fluorescence imaging is significantly more cost-efficient, and is particularly suitable for preliminary *in vivo* screening in small animals. Frangioni *et al*. reported that BAT could be imaged through tissue perfusion with a NIR dye. However, this method is also activation-dependent^48^. Rice *et al*. recently reported that micelle loaded with SRFluor680 dye could be used to visualize BAT^49^, but there was no application of the micelle for monitoring BAT mass change and “browning” process. In addition, Azhdarinia *et al*. reported that a peptide with a fluorescent tag that could be used for BAT imaging; however it is most likely not suitable for monitoring “browning”^50^. Reliable and easily synthesized NIR imaging probes to assess BAT mass and monitor browning are still very desirable.

Mouse interscapular BAT is an ideal target for Near-infrared fluorescence imaging due to the following reasons^41^: 1) the location of BAT in mice is unique since it is situated away from liver, heart, and stomach, and thus there is less signal interference from these large organs (Fig. 1a); 2) BAT location is shallow, which is perfect for the whole–body fluorescence imaging; 3) BAT is a whole mass organ; 4) BAT has a unique triangular physical shape which is easy to distinguish from other tissues (Fig. 1a). All of these features make BAT images extremely easy to recognize.

In this report, considering fluorescent compounds as “visible drugs”, we conducted a top-down imaging screening of 38 fluorescent dyes, and found that curcumin analogues CRANAD-2 and -3^51^,^52^,^53^ could highlight the interscapular BAT. However, the low BAT selectivity of CRANAD-2 and -3 indicated that further lead optimization was needed. To this end, we designed CRANAD-29 by increasing the hindrance of the lead probe to increase the facilitated uptake, and thus to increase the BAT selectivity. Our data indicated that CRANAD-29 had excellent BAT selectivity over WAT, and could be used for imaging BAT and monitoring browning of WAT.

## Results and Discussion

### Discovery of the lead probes through a top-down screening

**Figure 1:**
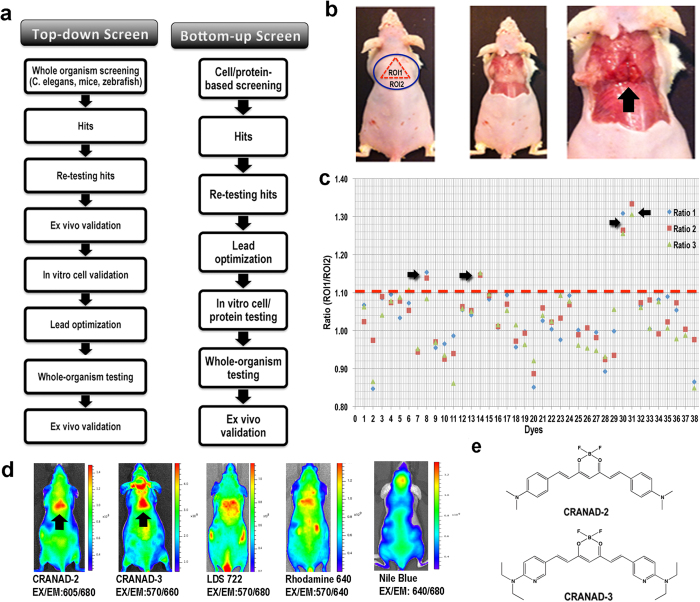
Top-down whole-organism screening of a library of 38 fluorescent dyes. (**a**) Schematic diagrams of a top-down (left) and a bottom-up screening (right). (**b**) Photographs of the locations of interscapular brown adipose tissue (BAT) and white adipose tissue (WAT). In the intact mouse (left), ROI_1_ (region of interest) in red dashed triangle represents BAT location, and ROI_2_ (blue circle) represents the areas of BAT, WAT, and adjacent non-adipose tissue area. The WAT that covers BAT can be seen after skin removal (middle), and the triangular shape of BAT (black arrow) can be clearly seen after the removal of skin and WAT (right). (**c**) Quantitative analysis of the top-down screening results. In the graph, each number represents a dye and each dye has three fluorescence signal readouts. The quantification was conducted using the ratio of the average fluorescence intensity of ROI_1_/ROI_2_, and the threshold was set at 1.10 (red dashed line). Four hits (black arrow) were selected for re-testing validation. (**d**) Representative near-infrared (NIR) fluorescence images of the screened dyes. CRANAD-2, CRANAD-3, LDS 722, and Rhodamine 640 showed apparent signal at the interscapular BAT area. Nile blue showed negative contrast for BAT. (e) Chemical structure of CRANAD-2 and -3.

Conventionally, a bottom-up cell- or protein-based testing/screening has been the most used approach for developing imaging probes^54^. However, this approach requires a known ligand for a specific biomarker as the starting point. Nonetheless, known imaging ligands for BAT mass are few^42^,^50^. To solve this problem, we conducted a top-down whole-organism screen (Fig. 1a) with fluorescent dyes, in which live animals were used as the screening platform instead of artificially engineered cells or isolated proteins. The top-down approach has already been used for drug screening in c. elegans and zebrafish^55^,^56^,^57^. However, to the best of our knowledge, this approach has not been investigated with mammals (nude mice). Considering imaging probes as “visible drugs”, this top-down approach could provide very clear information about the accumulation and location of the tested probes, and thus could effectively eliminate false positive hits.

We have collected 38 fluorescent dyes from commercial resources and from our laboratory. We selected dyes with the excitation range of 550–745 nm, the emission range of 600–840 nm, and with molecular weights less than 700 Da, except Cy5.5 (all of the dyes are listed at the end of SI Fig. 1 legend). The library was screened by intravenous tail injection of compounds into nude mice (10 μg/mouse). Each dye was imaged with three optimized excitation/emission filter-pairs and images at one hour after injection were acquired. As described in the Experimental Section, we screened the dyes based on the ROI (region of interest) ratios between BAT (ROI_1_) and BAT + WAT (ROI_2_) (Fig. 1b, left panel) using three excitation/emission pairs. The ratio was calculated based on the average intensities of ROI_1_ and ROI_2_ (R_(*BAT*)_ = ROI_1_/ROI_2_). We selected the dyes with the mean ratio R_(*BAT*)_ > 1.10 (n = 3) and with visible contrast around the interscapular BAT as preliminary positive hits (Fig. 1c).

By calculating the mean R_(*BAT*)_ of the dyes, we found that ratios of CRANAD-2 and CRANAD-3 were the highest (1.28 and 1.32 respectively) followed by LDS722 (1.13) and Rhodamine 640 (1.15) (Fig. 1c). By reviewing the visible contrast from the images, we found that CRANAD-2 and -3 (Fig. 1d, and chemical structure in Fig. 1e) provided the best contrast and clear triangular contour of interscapular BAT, while LDS722 and Rhodamine-640 showed weak contrast and no clear triangular contours (Fig. 1d). In addition, Phenoxazine 660 also showed a weak visible contrast, but its R_(*BAT*)_ was less than 1.10 (SI Fig. 1–3). Images of all 38 dyes tested *in vivo* are presented in SI Fig. 1.

### Negative contrast agents from the screening

Interestingly, when we carefully surveyed the images from the 38 tested dyes, we found that Nile blue provided a negative contrast at the interscapular BAT site (Fig. 1c,d) (R_(*BAT*)_ = 0.98). Further cell studies with differentiated 3T3-L1 cells suggested that Nile blue could label adipose cells and stain the oil droplets. However, we found that the emission of Nile blue was significantly blue-shifted, and the oil droplet staining could be observed from a 500–550 nm channel while Nile blue’s emission in PBS was around 700 nm. This result is consistent with reports, in which Nile blue and Nile red were used for staining oil droplets^58^,^59^. The significant emission blue-shift in oil droplets could explain why Nile blue provided a negative BAT contrast.

### Characterization of the lead probes

After the discovery of the lead probes CRANAD-2 and -3, we first estimated the probe’s selectivity for BAT over WAT. In this regard, we performed a stepwise dissection and imaging to track the source of the fluorescence contrast at the interscapular site. Skin and the thin WAT layer that covers BAT were first removed, and then BAT was dissected and NIR images were captured at each step. Once the skin and WAT were removed, both lead probes showed a significant decrease in signal, indicating that certain contrast originated from skin and WAT (SI Fig. 2a,b). However, fluorescence signals were higher for CRANAD-2 and -3 when BAT was present compared to the signals when it was dissected (SI Fig. 2a,b), indicating that the two probes were able to label BAT. We roughly estimated the signal contribution from BAT using the ratio of fluorescence signals acquired after (F_*(BAT)*_) and before (F_*(intact)*_) the first step of dissecting skin and WAT. Here we define BAT selectivity: S_BAT_ = F_(BAT)_/F_(intact)_, and F_(intact)_ is the fluorescent signal from the interscapular BAT area before dissection, and F_(BAT)_ is the fluorescent signal from BAT after the removal of skin and WAT that covers BAT. We found that S_BAT_ for CRANAD-2 was about 0.26, and 0.51 for CRANAD-3, indicating that the selectivity of these two probes for BAT was low. These data strongly suggested that further optimization of the lead probes was necessary. Other fluorescent dyes that showed weak BAT contrasts (Fig. 1c,d) were examined following the same dissection procedure as well; however our data showed that the weak signals did not originated from BAT, suggesting none of the dyes could be considered lead probes for BAT (SI Fig. 2c and SI Fig. 3).

We next investigated the time course of CRANAD-2 and -3 accumulation in BAT area. As seen in SI Fig. 4a–b, the uptake of CRANAD-2 reached its peak 120 minutes after injection, was detectable for at least 180 minutes, and was cleared by 720 minutes. Similarly, the signal of CRANAD-3 reached the uptake peak at around one hour, and then gradually washed out (SI Fig. 4c-d). Accumulation of CRANAD-2 and -3 in BAT cells was further confirmed with *ex vivo* histology of the dissected BAT slices. Fluorescence microscopy indicated that both CRANAD-2 and CRANAD-3 labeled oil droplets in brown adipose cells. The shape and size of CRANAD-2 and -3 stained oil droplets resembled those obtained with the gold standard H&E staining (SI Fig. 4e,f). However, we failed to obtain high-resolution images with the BAT tissue using a conventional microscope due to fast fading of the fluorescent signal and the merge of the oil droplets under excitation light.

**Figure 2:**
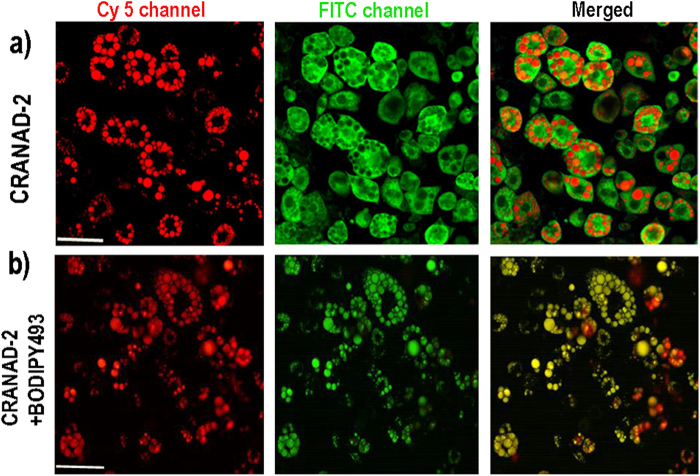
Two-photon cell imaging of 3T3-L1 adipose cells. (**a**) CRANAD-2 (left: red—CRANAD-2 signal, middle: green – autofluorescence of the cells, right: merged). (**b**) CRANDAD-2 + BODIPY493 (left: CRANAD-2 signal, middle: BODIPY493 signal, right: merged). Scale bar: 50 micron.

To further confirm the capacity of CRANAD-2 and CRANAD-3 for staining oil droplets, we incubated our compounds with differentiated 3T3-L1 adipose cells, which were induced from preadipocytes^7^,^8^,^60^. This cell line has been widely used for studies of both WAT^7^,^8^,^60^ and BAT, since these cells can be induced into both tissues^60^,^61^,^62^,^63^. In our report, 3T3-L1 cells are only used as an *in vitro* model for confirming staining of oil droplets and for limited uptake mechanism studies. Live cell confocal images indicated that the two probes were able to clearly label oil droplets in BAT cells, and that the droplet size was similar to that in our *ex vivo* studies with CRANAD-2 and -3 (data not shown). However, when we tried to obtain high magnification images (40X), we encountered same difficulties as *ex vivo* tissue imaging, due to the problems of fast signal fading and oil droplets merging. Fortunately, we found that a two-photon microscope was suitable for imaging CRANAD-2 in live cells. Two-photon imaging with 3T3-L1 adipose cells clearly demonstrated that CRANAD-2 accumulated in the oil droplets (Fig. 2a). For further validation, we used BODIPY493/505, a standard fluorescent dye for oil droplets^60^, for co-staining live adipose cells with CRANAD-2. We achieved an excellent co-localization of the two dyes in oil droplets (Fig. 2b). CRANAD-3 showed similar results (data not shown). Taken together, our *ex vivo* and *in vitro* cell imaging confirmed that the lead probe CRANAD-2 accumulated in oil droplets.

### Optimization of the lead probes

Although CRANAD-2 was able to provide a certain contrast for BAT *in vivo*, our stepwise dissection results indicated that their selectivity for BAT was poor (SI Fig. 2), which was probably due to the fast simple diffusion. In addition, their excitation and emission wavelengths were still shorter than that of an ideal NIR imaging probe (both excitation and emission >640 nm are ideal)^64^. To optimize the lead compounds, we first conducted limited uptake mechanism studies with CRANAD-2 (see details in the Supplemental information section, and SI Fig. 5, and SI Fig. 6), and found that the uptake of CRANAD-2 was probably a combination of fast simple diffusion and facilitated transporting via CD36/FAT (fatty acid translocase). For lead probe optimization, we aimed to extend their excitation/emission and reduce the fast simple diffusion, thereby increasing the facilitated uptake and BAT selectivity of the designed probe.

For small molecules, hydrophobicity and hindrance are two important factors for facilitated diffusion across the cell membrane^65^,^66^. The lower hydrophobicity, the higher possibility of facilitated diffusion the molecule has. Compared to CRANAD-2, CRANAD-3 showed higher BAT selectivity (S_BAT,_ 0.51 vs 0.26). Obviously, CRANAD-3 has lower hydrophobicity than CRANAD-2, and this could probably explain the better BAT selectivity of CRANAD-3. To further optimize CRANAD-3, we synthesized its analogues CRANAD-19, and -22, which have a polar morpholine moiety. CRANAD-22 could highlight BAT *in vivo* in mice (SI Fig. 7a); however the BAT selectivity of CRANAD-22 was not improved (data not shown), indicating lead optimization based on CRANAD-3 failed.

**Figure 3:**
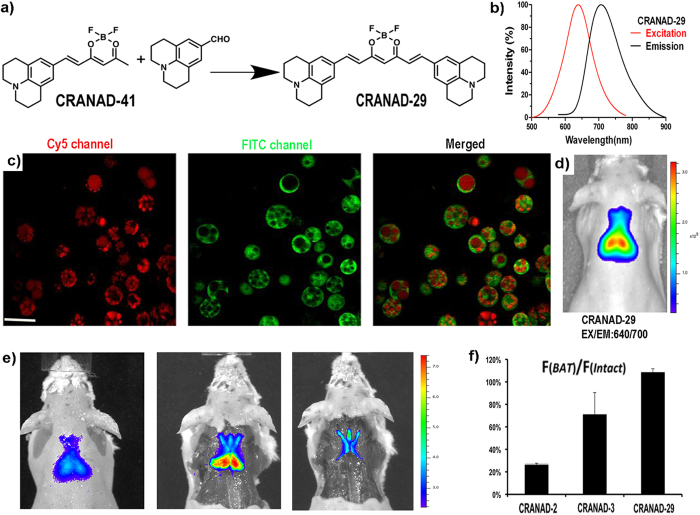
Lead optimization and *in vivo* NIR imaging. (**a**) The structure and synthesis of CRANAD-29. (**b**) Excitation and emission spectra of CRANAD-29. (**c**) Two-photon microscopic imaging of 3T3-L1 cells with CRANAD-29 (left: red—CRANAD-29 signal, middle: green – autofluorescence of the cells, right: merged). The images clearly demonstrated that CRANAD-29 was accumulated in the oil droplets. Scale bar: 50 micron. (**d**) Representative *in vivo* NIR image of a CRANAD-29 injected mouse. The two lobes of the interscapular BAT can been clearly seen. (**e**) Stepwise dissection and validation of BAT signal after CRANAD-29 injection. There was no significant signal decrease after skin and WAT removal (middle), while the signal disappeared after BAT removal (right). (**f**) Comparison of F_(BAT)_/F_(intact)_ ratio for CRANAD-2, -3, and -29. The ratio for CRANAD-29 was much higher than that for CRANAD-2 and -3, indicating that the selectivity of BAT over WAT was significantly improved after the lead optimization.

The uptake curve of CRANAD-2 in 3T3-L1 cells showed a very quick uptake phase within one minute (SI Fig. 5a, right panel), which was probably due to the fast simple diffusion that significantly contributed to the poor selectivity of CRANAD-2 for BAT *in vivo*. It is known that bulky compound have less tendency for simple diffusion^65^,^66^,^67^,^68^. We hypothesized that the poor BAT selectivity of CRANAD-2 could be significantly improved via increasing the molecule’s hindrance. It has been reported that Julolidine rings could significantly increase the hindrance^69^. In addition, replacement of N,N-dialkylamino-phenyl moiety with a julolidine ring could extend excitation and emission wavelengths^69^. Based on these facts and the structure of CRANAD-2, we designed CRANAD-29 to minimize simple diffusion via increasing the hindrance of the molecule, and to extend the excitation/emission (Fig. 3a). CRANAD-29 was synthesized through a two-step procedure (Fig. 3a), in which an intermediate CRANAD-41 was purified first and then reacted with the corresponding aldehyde to give CRANAD-29. We found that this probe had significantly longer excitation (640 nm) and emission (720 nm) (Fig. 3b). Two-photon imaging with differentiated 3T3-L1 cells clearly indicated that CRANAD-29 accumulated in the oil droplets (Fig. 3c). We compared the uptake of CRANAD-29 in 3T3-L1 cells before and after differentiation. As expected, CRANAD-29 showed no apparent uptake in undifferentiated cells over the time course of the study (SI Fig. 8a, left panel), indicating no significant simple diffusion. Indeed, its uptake in differentiated cells was much slower than CRANAD-2 and reached its plateau around 90 minutes (SI Fig. 8a, middle panel), indicating the contribution from simple diffusion was minimized. It is known that facilitated diffusion can be significantly inhibited when the cells were fixed^70^. Indeed, the uptake of CRANAD-29 was reduced 70% when the differentiated 3T3-L1 cells were fixed with glutaraldehyde (SI Fig. 8a, right panel), suggesting CRANAD-29 was primarily transported via the facilitated transporting. Moreover, similar to CRANAD-2, the uptake of CRANAD-29 could be significantly reduced by triglyceride, indicating that CRANAD-29 transport was probably related to CD36 (SI Fig. 8b,c). Additionally, we found that the uptake of CRANAD-29 could be significantly reduced by Hexarelin, a CD36 specific ligand^71^,^72^ (SI Fig. 8d). This data further indicated that the uptake of CRANAD-29 could be related to CD36 facilitated transporting. CD36 is highly expressed on the surface of adipose cells and endothelial cells of capillaries in adipose tissue^73^,^74^,^75^,^76^. BAT is highly vascularized and has abundant capillaries, while the number of vessels and capillaries in WAT is significantly lower. This means that a higher CD36 specificity of CRANAD-29 in combination with lower fast simple diffusion could lead to a higher BAT selectivity for this probe.

Although our data indicated that the uptake of CRANDAD-2 and -29 could be significantly suppressed by triglyceride, and the facilitated diffusion via CD36 was likely involved, other transporters such as FABP4, GPIHBP1, Mfge8, and caveolin-1 could also be engaged in this process^77^,^78^,^79^.

As expected, *in vivo* imaging with CRANAD-29 showed excellent contrast and a very clear contour of BAT at the interscapular site (Fig. 3d). In addition, the two lobes of the interscapular BAT can be clearly seen. Remarkably, after analyzing the fluorescence intensity of the signals from the step-wise dissection experiment, we found that CRANAD-29 had excellent selectivity towards BAT over WAT (Fig. 3e). As predicted, CRANAD-29 showed much better BAT selectivity over WAT than CRANAD-2 and -3 (Fig. 3f). We noted that the fluorescence signal after skin and WAT removal was even higher than that of the intact animal (Fig. 3e,f), which is probably due to less light scattering and higher BAT exposure once skin and WAT were removed. In addition, we observed a “spider” shape that was highlighted by a fluorescent signal after the BAT removal (Fig. 3e, right panel). However, it is not clear whether the “spider” shape consisted of stretched brown adipose tissue.

To further validate the capacity of CRANAD-29 for BAT labeling, *ex vivo* NIR imaging and histological microscopic imaging with CRANAD-29 was conducted. NIR imaging showed that CRANAD-29 had clear BAT selectivity over WAT (SI Fig. 9a). Microscopic images indicated that CRANAD-29 was capable of labeling BAT cells and each cell contained multiple CRANAD-29-stained oil droplets (SI Fig. 9b). Gold standard H&E staining also provided similar images (SI Fig. 9c). A time course study indicated that the uptake of CRANAD-29 reached its plateau approximately 2 hours after probe injection (SI Fig. 9d). We also optimized the injection dose of CRANAD-29, and found that the fluorescence signal reached its plateau at 0.4 mg/kg (SI Fig. 10). In the following studies, a 0.4 mg/kg dose was used. In addition, bio-distribution studies of CRANAD-29 indicated that interscapular BAT had the highest uptake (SI Fig. 11a).

For the above reasons, we selected CRANAD-29 as the imaging probe for our proof-of-concept applications described below.

### Imaging applications of CRANAD-29

**Figure 4:**
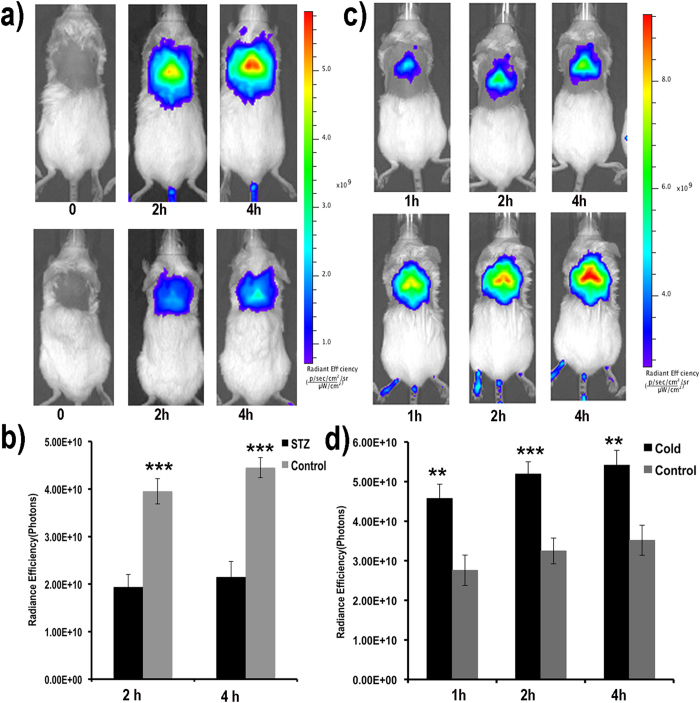
Application of CRANAD-29 for monitoring BAT mass change and BAT activation. (**a**) Representative NIR images of control mice (top) and mice after STZ-treatment (bottom). (**b**) Quantitative analysis of the fluorescence signals in (**a**). The signal from STZ-induced diabetic mice was significantly lower than that from control mice. (**c**) Representative NIR images of control mice (top) and mice after cold exposure (bottom). (d) Quantitative analysis of the fluorescence signals in (**c**). The signal from mice exposed to cold stress was significantly higher than that of control mice, indicating that CRANAD-29 could be used for monitoring BAT activation.

To demonstrate the feasibility of CRANAD-29 for monitoring BAT mass change during diabetes development, we utilized a widely used streptozotocin (STZ)-induced type 1 diabetes model. Several studies have reported that BAT mass is dramatically reduced after STZ treatment^18^,^80^,^81^,^82^. We also found that after seven days of STZ treatment, interscapular BAT was significantly diminished, and this change was evident even from the regular photographic images (SI Fig. 11b). After CRANAD-29 injection, we found that the fluorescence signal from the diabetic mice was significantly lower than that from the control group, reflecting significant BAT mass decrease after STZ treatment (Fig. 4a-b). We found an excellent correlation between dissected BAT mass and fluorescence signal in STZ-treated animals and normal animals (SI Fig. 11c). These results indicate that fluorescence imaging using CRANAD-29 could be used to report on the relative change of BAT mass.

BAT could be activated under various conditions, including short cold exposure^9^,^83^. In this report, we investigated whether CRANAD-29 could be used to monitor BAT activation under these conditions. Animals that were subjected to 2 hours of cold exposure displayed 1.65-, 1.59-, and 1.53-fold higher signal after CRANAD-29 administration than the control group at 1-, 2-, and 4-hours post probe injection, indicating that CRANAD-29 could be used for monitoring BAT activation (Fig. 4c,d). Although research groups used different protocols for cold stimulating BAT, the detected increase after the cold activation from CRANAD-29 was higher than other NIRF dyes. For instance, IR786 showed only about a 1.4-fold increase^48^, while CRANAD-29 showed a 1.65-fold increase. Micelle containing SRFluoro680 showed insignificant difference before and after the cold stimulation. However, it is not feasible to compare our NIRF results with PET imaging with 18F-FDG because different groups reported significantly different increases after cold treatment^39^,^84^.

**Figure 5:**
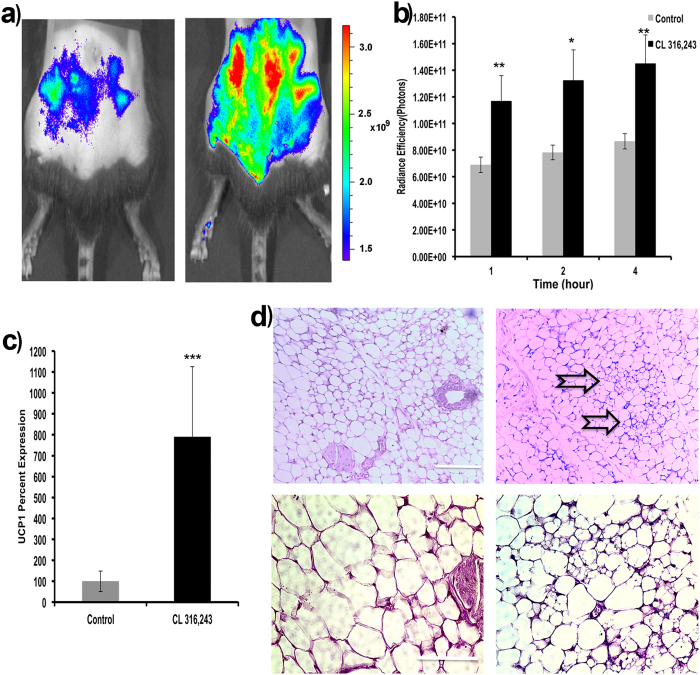
Application of CRANAD-29 for monitoring the browning of sWAT. (**a**) Representative NIR images of a control mouse (left) and a mouse after 12-day CL 316,243 treatment (right) at 4 hours post CRANAD-29 injection. (**b**) Quantitative analysis of the fluorescence signals at 1, 2, and 4 hours post CRANAD-29 injection (Note that different ROI sizes were used for sWAT in Fig. 5 and iBAT in Fig. 4). (**c**) UCP-1 expression in sWATs of CL 316,243 treated mice and control mice. (**d**) H&E staining of sWAT slices of a CL 316,243-treated mouse (right) and a control mouse (left). Black arrows indicated the areas containing multilocular fat cells. Top row: low resolution (10 x), scale bar: 200 micron, bottom row: high resolution (40 x), scale bar: 100 micron.

Browning of white adipose tissue could be achieved through several approaches, including small molecule stimulation such as β3-adrenoceptor agonist (CL316,243), PPAR-gamma ligand (rosiglitazone), treatment with hormones and cytokines, and genetic manipulation^19^,^20^,^21^,^26^,^29^,^85^. It has been reported that CL316,243 could induced acute BAT activation with short treatment^48^, and browning with chronic treatment^19^,^20^,^21^,^26^,^29^,^85^. As a proof-of-concept, in this report, we used CL316,243 to treat mice for establishing the browning model. After 12 days of CL316,243 injection, we found the signal of CRANAD-29 was significantly higher around the inguinal subcutaneous WAT in the treated group than that of the control group (Fig. 5a,b). This result was coherent with UCP-1 expression and H&E staining results (Fig. 5c,d). The UCP-1 mRNA level was 7.9-fold higher in the treated mice than that of the control group (Fig. 5c). H&E staining showed cells with multilocular lipid droplets in the sWAT slice of the treated mice (Fig. 5d). In addition, we observed a slightly higher fluorescence signal from the interscapular BAT area in the treated group than that of the control group (SI Fig. 12). Our data suggested that CRANAD-29 could be used for monitoring the browning process.

It is known that short treatment with stimulators such as Norepinephrine and cold always induce BAT activation, while BAT mass change and browning are normally due to chronic drug treatment such as CL316,243. Therefore, although CRANAD-29 can be used for monitoring both activation and BAT mass change, it should have the capability to differentiate these effects under proper experimental conditions. Our data indicated that CRANAD-29 could be used for monitoring browning; however, the exact mechanism is not clear. Recent evidences showed that browning of WAT could induce vascularization in WAT^1^,^86^,^87^,^88^, and this could be one of the possible explanations for CRANAD-29′s capability of monitoring browning.

### Summary

In summary, we demonstrated the utility of curcumin-based probes for *in vivo* NIR fluorescence imaging of BAT and browning of sWAT. Through the optimization of the lead probes via increasing the stereo hindrance, we have successfully devised CRANAD-29 as a highly BAT selective imaging probe, which could be used for monitoring BAT mass change, BAT activation, and browning of sWAT. Since these probes could be easily adapted for labeling with radioactive isotopes, clinical translation of our approach is feasible and will be pursued in the near future.

## Methods

Fluorescent dyes were purchased from Exciton Inc. (Dayton, OH) and Invitrogen. The reagents used for the synthesis were purchased from Aldrich and used without further purification. Column chromatography was performed on silica gel (SiliCycle Inc., 60 Å, 40–63 mm) slurry packed into glass columns. ^1^H, ^13^C NMR spectra were recorded at 500 MHz and 125 MHz respectively, and reported in ppm downfield from tetramethylsilane. Fluorescence studies were carried out using a F-4500 Fluorescence Spectrophotometer (Hitachi). Mass spectra were obtained at Harvard University, Department of Chemistry Instrumentation Facility. All animal experimental procedures were approved by the Institutional Animal Care and Use Committee (IACUC) at Massachusetts General Hospital, and carried out in accordance with the approved guidelines. Microscopic images were acquired with Nikon Eclipse 50i microscope. Two-photon imaging was performed on a two-photon microscope (Prairie Technologies) equipped with a 20x water immersion objective (N.A. 1.0, Zeiss). *In vivo* NIR imaging was performed using the IVIS^®^Spectrum animal imaging system (Caliper LifeSciences, Perkin Elmer, Hopkinton, MA), and data analysis was conducted using LivingImage^®^ 4.2.1 software.

### Top-down whole organism screening

Nude mice (nu/nu COX7) were purchased from Massachusetts General Hospital and Balb/c mice were purchased from Jackson Laboratory. Dye stock solutions were prepared in DMSO. The final injection solutions (0.1 mg/ml) were freshly prepared in a mixed solution of 15% DMSO, 15% Cremophor EL, and 70% saline. Nude mice were injected with a 100 μL dye solution via tail vein. For each imaging session, mice were anesthetized with a mixture of oxygen and isoflurane for 5 minutes, and then positioned in the imaging chamber of IVIS Spectrum whole body imaging system (PerkinElmer, Hopkinton, MA). Images were acquired at one-hour post injection using sequence imaging with three excitation/emission filter pairs for each dye.

### Hits selection

Hits from cell- or protein-based screening are usually selected by setting a certain threshold. In the case of our top-down imaging screening, we used two methods (threshold and visible contrast) to select hits. We first selected three images obtained with three excitation/emission pairs for each dye. These ex/em pairs were selected from the closest excitation and emission wavelengths of the tested dyes. For instance, 605/660 nm, 605/680 nm, and 640/680 nm were selected for image acquisition of Nile blue (625/660 nm). The quantification of contrast for each image was calculated as a ratio of two ROIs (region of interest). ROI_1_ reflected the average signal from interscapular BAT and ROI_2_ was the average signal of the area that included BAT and white adipose tissue (WAT) around the interscapular site (Fig. 1a, left). We used the averaged fluorescent signal of ROI instead of the total signal, because the total signal depends on the area size of ROI, whereas the averaged signal reflects the density of signal. The ratio of ROI_1_/ROI_2_ (R_*(BAT)*_) could reflect the signal/noise ratio of the tested probe and could also reflect the selectivity of the probe for BAT over WAT. For each dye, three ROI_1_/ROI_2_ ratios obtained with the three filter pairs were calculated. The mean of the ratios was used as the threshold. If the mean ratio was >1.10, the dye was selected as a potential positive hit (Fig. 1c). Since the contrast of a dye in the top-down screening is in the visible range, we also tested the visible contrast around the interscapular BAT area for the hits with a mean R_*(BAT)*_ >1.10. If both mean ratio and the visible contrast were positive, then the hit was subjected to the re-testing procedure to validate its reliability as described below.

### Re-testing of the hits and stepwise dissection

CRANAD-2, -3, LDS 722 and Rhodamine 640 were subjected to re-test by repeating the above imaging procedure. Images were acquired one hour after probe injection. Animals were then sacrificed, followed by a two-step dissection. The first step was to remove skin and WAT, and the second step was to remove BAT at the interscapular site. NIR images were captured at each step.

### *Ex vivo* histological imaging

The above-dissected BAT was fixed with 4% formalin at 4°C overnight, and then embedded in OCT. BAT tissue was cut into 7 micron thick slices, washed with PBS buffer, and co-stained with DAPI (Vectra Shield, Vector Lab, Burlingame, CA). Images were acquired with fast exposure to capture the dye signal in the oil droplets. Note, we experienced some difficulties in capturing images for high magnification, probably due to very fast bleaching of the dye under microscopic imaging and the merging of the oil droplets to form bigger oil droplets.

### Two-photon imaging for 3T3-L1 cells

Fibroblast 3T3-L1 cells were differentiated following the protocol provided by Zenbio, Inc. (protocol No. ZBM0009.02). Ten microliters of CRANAD-2 (250 μM in DMSO) was added to the differentiated cells (1.0 ml). Cells were imaged after 10 min of incubation. A 940 nm laser was first used to capture an image for CRANAD-2 in 570-620 nm channel (Prairie Two-photon microscope, Middleton, WI). Next, the laser was tuned to 750 nm to capture autofluorescence of the cells to outline cell morphology.

For triglyceride competition, stock solutions of CRANAD-2 (250 μM) and triglyceride (5 mM) in DMSO were prepared. Before imaging, 10 μl of the stock solution was added to the cells.

### IVIS imaging of 3T3-L1 cells with CRANAD-2

To a 6-well plate seeded with preadipocytes or differentiated adipocytes, a 10μL solution of CRANAD-2 (250 μM in DMSO) was added. The plate was subjected to imaging using IVIS imaging system with Ex = 605 nm, Em = 660 nm before and after addition of CRANAD-2. It was not necessary to wash or fix the cells before each imaging session, because CRANAD-2 or its analogues showed weak fluorescence in the medium, but their fluorescence was significantly increased once they entered into the hydrophobic oil droplets. For triglyceride competition, the same protocol for two-photon imaging was used. The images were acquired at 20 min after addition of CRANAD-2. Studies were performed in triplicate.

### Synthesis of CRANAD-19, -22, -29, and -41

These compounds were prepared according to our previous reported procedures^51^,^89^. Briefly, the 2,2-difluoro-1,3-dioxaboryl-pentadione crystals (0.075 g, 0.5 mmol) were dissolved in acetonitrile (1.5 ml), followed by the additions of acetic acid (0.1 ml), tetrahydroisoquinoline (0.02 ml, 0.15 mmol), and aromatic aldehyde (1.0 mmol). The resulted solution was stirred at 60 °C overnight. A black residue obtained after removing the solvent was subjected to flash column chromatography to give a dark powder.

### CRANAD-19

yield 21.5%. ^1^H NMR (CDCl_3_) δ(ppm) 2.09 (s, 3H), 3.61 (t, 8H, J = 4.5 Hz), 3.75 (t, 3H, J = 4.5 Hz), 6.58 (d, 2H, J = 10.5 Hz), 6.78 (d, 2H, J = 15.5 Hz), 7.70 (dd, 2H, J = 10.5, 2.0 Hz), 7.92 (d, 2H, J = 15.5 Hz), 8.33 (d, 2H, J = 2.0 Hz); ^13^C NMR (d-DMSO) δ(ppm) 11.31, 45.12, 66.33, 107.23, 107.61, 114.77, 120.50, 137.15, 144.64, 153.14, 159.88, 176.63; ^19^F NMR (CDCl_3_) δ(ppm) 143.72, 143.78; ESI-MS (M^+^) m/z = 511.3. **CRANAD-22:** Yield 28.9%. ^1^H NMR (CDCl_3_) δ(ppm) 1.17 (t, 6H, J = 7.5 Hz), 3.54 (q, 4H, J = 7.5 Hz), 3.60 (t, 4H, J = 5.0 Hz), 3.74 (t, 4H, J = 5.0 Hz), 5.85 (s, 1H), 6.37 (d, 1H, J = 16.0Hz), 6.42 (d, 1H, J = 16.0 Hz), 6.46 (d, 1H, J = 8.5 Hz), 6.58 (d, 1H, J = 8.5 Hz), 7.61 (d, 1H, J = 7.5 Hz), 7.66 (d, 1H, J = 7.5 Hz), 7.84 (d, 1H, J = 16.0 Hz), 7.87 (d, 1H, J = 16.0 Hz), 8.30 (s, 2H); ^13^C NMR (CDCl_3_) δ(ppm) 12.92, 43.14, 44.98, 66,56, 101.25, 106.21, 106.55, 114.81, 116.85, 118.18, 120.02, 135.66, 136.00, 143.04, 145.03, 151.67, 153.14, 158.63, 159.74, 177.57, 178.80 ; ^19^F NMR (CDCl_3_) δ(ppm) 141.80; ESI-MS (M^+^) m/z = 483.2. **CRANAD-29:** This compound was synthesized from CRANAD-41, yield 9.0% (based on CRANAD-41). ^1^H NMR (CDCl_3_) δ(ppm) 1.89 (quit, 8H, J = 6.0 Hz), 2.67 (t, 8H, J = 6.0 Hz), 3.21 (t, 8H, J = 5.5 Hz), 5.71 (s, 1H), 6.28 (d, 2H, J = 15.5 Hz), 6.98 (s, 4H), 7.75 (d, 2H, J = 15.5 Hz); ^13^C NMR (CDCl_3_) δ(ppm) 21.37, 27.65, 50.09, 100.66, 113.82, 121.13, 121.54, 129.03, 146.20, 146.54, 176.90; ^19^F NMR (CDCl_3_) δ(ppm) 142.87, 142.93; ESI-MS (M^+^) m/z = 515.3. **CRANAD-41:** yield 37%. ^1^H NMR (CDCl_3_) δ(ppm) 1.89 (m, 4H), 2.14 (s, 3H), 2.66 (t, 4H, J = 6.0 Hz), 3.24 (t, 4H, J = 6.0 Hz), 5.72 (s, 1H), 6.20(d, 1H, J = 15.0 Hz), 7.00 (s, 2H), 7.86 (d, 1H, J = 15.0 Hz); ^13^C NMR (CDCl_3_) δ(ppm) 21.18, 23.74, 27.60, 50.18, 100.10, 111.47, 120.76, 121.24, 129.95, 147.35, 150.49, 180.24, 185.20 ; ^19^F NMR (CDCl_3_) δ(ppm) 141.12, 141.18; ESI-MS (2M-2H + Na) m/z = 685.3.

### IVIS imaging of 3T3-L1 cells with CRANAD-29

To a 6-well plate seeded with preadipocytes or differentiated adipocytes, a 10 μL solution of CRANAD-29 (250 μM in DMSO) was added. The plate was subjected to imaging using IVIS imaging system with Ex = 640 nm, Em = 700 nm before and after addition of CRANAD-29. For triglyceride competition and Hexarelin inhibition, the similar protocol for CRANAD-2 imaging was used. The final concentration of hexarelin was 12.5 μM, and the images were acquired at 3 hours after addition of CRANAD-29 and triglyceride/Hexarelin. Studies were performed in triplicate.

### Bio-distribution study

For the bio-distribution study, Balb/c mice (n = 5) were injected with CRANAD-29 intravenously. BAT and other major organs/tissues were dissected at 4 hours post injection, which were then weighed and subjected to NIR imaging. Fluorescence intensities were normalized to the weight of organ/tissue.

### Monitoring BAT mass change in STZ-induced diabetic model with CRANAD-29

Two-month old Balb/c mice (n = 5) were injected with STZ (80 mg/kg) for 7 days. When blood glucose levels reached 250 mg/dL for two consecutive days, the mice were subjected to NIR imaging with CRANAD-29. Before imaging, the fur around the interscapular area was removed. Images were acquired pre-injection, as well as 2- and 4-hours after intravenous injection. After imaging, the mice were sacrificed, BAT from both groups was dissected and weighed, and a linear correlation was established between *in vivo* fluorescence signal and weight of the dissected BATs. The same imaging procedure was conducted with the control group that was injected with saline of the same volume for 7 days (n = 5).

### Monitoring BAT activation under short cold exposure with CRANAD-29

Two-month old balb/c mice (n = 5) were placed in a 4 °C cold room for 2 hours before intravenous injection of CRANAD-29. Images were acquired at 1-, 2-, 4-hours after probe injection, and mice were placed in the cold room between imaging sessions. Control mice (n = 5) were placed in a 25 °C room. Images were captured at the same time points after CRANAD-29 injection.

### Monitoring browning of sWAT with CRANAD-29

Two-month old C57BL6 mice (n = 5) were intraperitoneally injected with CL 316,243 (100 uL, 1.0 mg/kg) for 12 days, and the control mice (n = 5) were intraperitoneally injected with saline. On the 13^th^ day, both groups were imaged after intravenous injection of CRANAD-29. Images were acquired at 1-, 2-, 4-hours after the probe injection. After the last imaging session, inguinal WATs were dissected and subjected to qPCR for UCP-1 expression and H&E staining.

## Additional Information

**How to cite this article**: Zhang, X. *et al*. Curcumin analogues as selective fluorescence imaging probes for brown adipose tissue and monitoring browning. *Sci. Rep*. **5**, 13116; doi: 10.1038/srep13116 (2015).

## Supplementary Material

Supplementary Information

## References

[b1] Cannon B. & Nedergaard J. Brown adipose tissue: function and physiological significance. Physiol. Rev. 84, 277–359 (2004). 14715917 10.1152/physrev.00015.2003

[b2] Richard D. & Picard F. Brown fat biology and thermogenesis. Frontiers in bioscience: a journal and virtual library 16, 1233–1260 (2011). 10.2741/3786 21196229

[b3] Ouellet V. . Brown adipose tissue oxidative metabolism contributes to energy expenditure during acute cold exposure in humans. The Journal of clinical investigation 122, 545–552 (2012). 22269323 10.1172/JCI60433 PMC3266793

[b4] Cypess A. M. . Identification and importance of brown adipose tissue in adult humans. The New England journal of medicine 360, 1509–1517 (2009). 19357406 10.1056/NEJMoa0810780 PMC2859951

[b5] Nedergaard J., Bengtsson T. & Cannon B. Unexpected evidence for active brown adipose tissue in adult humans. American journal of physiology. Endocrinology and metabolism 293, E444–452 (2007). 17473055 10.1152/ajpendo.00691.2006

[b6] Tran T. T. & Kahn C. R. Transplantation of adipose tissue and stem cells: role in metabolism and disease. Nature reviews. Endocrinology 6, 195–213 (2010). 10.1038/nrendo.2010.20 PMC4362513 20195269

[b7] Tseng Y. H. . New role of bone morphogenetic protein 7 in brown adipogenesis and energy expenditure. Nature 454, 1000–1004 (2008). 18719589 10.1038/nature07221 PMC2745972

[b8] Zhang H. . Cross talk between insulin and bone morphogenetic protein signaling systems in brown adipogenesis. Mol. Cell. Biol. 30, 4224–4233 (2010). 20584981 10.1128/MCB.00363-10 PMC2937545

[b9] van Marken Lichtenbelt W. D. . Cold-activated brown adipose tissue in healthy men. The New England journal of medicine 360, 1500–1508 (2009). 19357405 10.1056/NEJMoa0808718

[b10] Virtanen K. A. . Functional brown adipose tissue in healthy adults. The New England journal of medicine 360, 1518–1525 (2009). 19357407 10.1056/NEJMoa0808949

[b11] Hany T. F. . Brown adipose tissue: a factor to consider in symmetrical tracer uptake in the neck and upper chest region. Eur. J. Nucl. Med. Mol. Imag. 29, 1393–1398 (2002). 10.1007/s00259-002-0902-6 12271425

[b12] Saito M. . High incidence of metabolically active brown adipose tissue in healthy adult humans: effects of cold exposure and adiposity. Diabetes 58, 1526–1531 (2009). 19401428 10.2337/db09-0530 PMC2699872

[b13] Cohade C., Osman M., Pannu H. K. & Wahl R. L. Uptake in supraclavicular area fat (“USA-Fat”): description on 18F-FDG PET/CT. Journal of nuclear medicine: official publication, Society of Nuclear Medicine 44, 170–176 (2003). 12571205

[b14] Zingaretti M. C. . The presence of UCP1 demonstrates that metabolically active adipose tissue in the neck of adult humans truly represents brown adipose tissue. FASEB journal: official publication of the Federation of American Societies for Experimental Biology 23, 3113–3120 (2009). 19417078 10.1096/fj.09-133546

[b15] Schulz T. J. & Tseng Y. H. Brown adipose tissue: development, metabolism and beyond. The Biochemical journal 453, 167–178 (2013). 23805974 10.1042/BJ20130457 PMC3887508

[b16] Yoneshiro T. . Age-related decrease in cold-activated brown adipose tissue and accumulation of body fat in healthy humans. Obesity 19, 1755–1760 (2011). 21566561 10.1038/oby.2011.125

[b17] Boss O. & Farmer S. R. Recruitment of brown adipose tissue as a therapy for obesity-associated diseases. Frontiers in endocrinology 3, 14 (2012). 22654854 10.3389/fendo.2012.00014 PMC3356088

[b18] Gunawardana S. C. & Piston D. W. Reversal of type 1 diabetes in mice by brown adipose tissue transplant. Diabetes 61, 674–682 (2012). 22315305 10.2337/db11-0510 PMC3282804

[b19] Kajimura S. . Regulation of the brown and white fat gene programs through a PRDM16/CtBP transcriptional complex. Genes Dev. 22, 1397–1409 (2008). 18483224 10.1101/gad.1666108 PMC2377193

[b20] Farmer S. R. Molecular determinants of brown adipocyte formation and function. Genes Dev. 22, 1269–1275 (2008). 18483216 10.1101/gad.1681308 PMC2732411

[b21] Wilson-Fritch L. . Mitochondrial remodeling in adipose tissue associated with obesity and treatment with rosiglitazone. The Journal of clinical investigation 114, 1281–1289 (2004). 15520860 10.1172/JCI21752 PMC524228

[b22] Qiang L. . Brown remodeling of white adipose tissue by SirT1-dependent deacetylation of Ppargamma. Cell 150, 620–632 (2012). 22863012 10.1016/j.cell.2012.06.027 PMC3413172

[b23] Wu J. . Beige adipocytes are a distinct type of thermogenic fat cell in mouse and human. Cell 150, 366–376 (2012). 22796012 10.1016/j.cell.2012.05.016 PMC3402601

[b24] Rao R. R. . Meteorin-like Is a Hormone that Regulates Immune-Adipose Interactions to Increase Beige Fat Thermogenesis. Cell 157, 1279–1291 (2014). 24906147 10.1016/j.cell.2014.03.065 PMC4131287

[b25] Wu J., Cohen P. & Spiegelman B. M. Adaptive thermogenesis in adipocytes: is beige the new brown? Genes Dev. 27, 234–250 (2013). 23388824 10.1101/gad.211649.112 PMC3576510

[b26] Schulz T. J. . Brown-fat paucity due to impaired BMP signalling induces compensatory browning of white fat. Nature 495, 379–383 (2013). 23485971 10.1038/nature11943 PMC3623555

[b27] Xu X. . Exercise ameliorates high-fat diet-induced metabolic and vascular dysfunction, and increases adipocyte progenitor cell population in brown adipose tissue. *American journal of physiology*. Regulatory, integrative and comparative physiology 300, R1115–1125 (2011). 10.1152/ajpregu.00806.2010 PMC3094041 21368268

[b28] Bostrom P. . A PGC1-alpha-dependent myokine that drives brown-fat-like development of white fat and thermogenesis. Nature 481, 463–468 (2012). 22237023 10.1038/nature10777 PMC3522098

[b29] Himms-Hagen J. . Multilocular fat cells in WAT of CL-316243-treated rats derive directly from white adipocytes. American journal of physiology. Cell physiology 279, C670–681 (2000). 10942717 10.1152/ajpcell.2000.279.3.C670

[b30] Chen Y. . miR-155 regulates differentiation of brown and beige adipocytes via a bistable circuit. Nature communications 4, 1769 (2013). 10.1038/ncomms2742 PMC3644088 23612310

[b31] Liu W. . miR-133a Regulates Adipocyte Browning *In Vivo*. PLoS Genet. 9, e1003626 (2013). 23874225 10.1371/journal.pgen.1003626 PMC3708806

[b32] Mattson M. P. Perspective: Does brown fat protect against diseases of aging? Ageing research reviews 9, 69–76 (2010). 19969105 10.1016/j.arr.2009.11.004 PMC2818667

[b33] Pfannenberg C. . Impact of age on the relationships of brown adipose tissue with sex and adiposity in humans. Diabetes 59, 1789–1793 (2010). 20357363 10.2337/db10-0004 PMC2889780

[b34] Nagajyothi F. . Response of adipose tissue to early infection with Trypanosoma cruzi (Brazil strain). The Journal of infectious diseases 205, 830–840 (2012). 22293433 10.1093/infdis/jir840 PMC3274374

[b35] Herrero L., Shapiro H., Nayer A., Lee J. & Shoelson S. E. Inflammation and adipose tissue macrophages in lipodystrophic mice. Proceedings of the National Academy of Sciences of the United States of America 107, 240–245 (2010). 20007767 10.1073/pnas.0905310107 PMC2806777

[b36] Basu S. Functional imaging of brown adipose tissue with PET: can this provide new insights into the pathophysiology of obesity and thereby direct antiobesity strategies? Nuclear medicine communications 29, 931–933 (2008). 18836369 10.1097/MNM.0b013e328310af46

[b37] Bartelt A. . Brown adipose tissue activity controls triglyceride clearance. Nat. Med. 17, 200–205 (2011). 21258337 10.1038/nm.2297

[b38] Aleo M. D. . Mechanism and implications of brown adipose tissue proliferation in rats and monkeys treated with the thiazolidinedione darglitazone, a potent peroxisome proliferator-activated receptor-gamma agonist. The Journal of pharmacology and experimental therapeutics 305, 1173–1182 (2003). 12626651 10.1124/jpet.102.042648

[b39] Wu C. . Brown adipose tissue can be activated or inhibited within an hour before 18F-FDG injection: a preliminary study with microPET. Journal of biomedicine & biotechnology 2011, 159834 (2011). 21541240 10.1155/2011/159834 PMC3085214

[b40] Tatsumi M. . Intense (18)F-FDG uptake in brown fat can be reduced pharmacologically. Journal of nuclear medicine: official publication, Society of Nuclear Medicine 45, 1189–1193 (2004). 15235065

[b41] Zhang X., Kuo C., Moore A. & Ran C. *In Vivo* Optical Imaging of Interscapular Brown Adipose Tissue with 18F-FDG via Cerenkov Luminescence Imaging. Plos One 8, e62007 (2013). 23637947 10.1371/journal.pone.0062007 PMC3634850

[b42] Madar I., Isoda T., Finley P., Angle J. & Wahl R. 18F-fluorobenzyl triphenyl phosphonium: a noninvasive sensor of brown adipose tissue thermogenesis. Journal of nuclear medicine: official publication, Society of Nuclear Medicine 52, 808–814 (2011). 10.2967/jnumed.110.084657 PMC4332805 21498536

[b43] Hu H. H., Smith D. L. Jr., Nayak K. S., Goran M. I. & Nagy T. R. Identification of brown adipose tissue in mice with fat-water IDEAL-MRI. Journal of magnetic resonance imaging: JMRI 31, 1195–1202 (2010). 20432356 10.1002/jmri.22162 PMC2924147

[b44] Chen Y. I. . Anatomical and Functional Assessment of Brown Adipose Tissue by Magnetic Resonance Imaging. Obesity 20, 1519–1526 (2012). 22343821 10.1038/oby.2012.22 PMC4383098

[b45] Khanna A. & Branca R. T. Detecting brown adipose tissue activity with BOLD MRI in mice. Magnetic resonance in medicine: official journal of the Society of Magnetic Resonance in Medicine/Society of Magnetic Resonance in Medicine 68, 1285–1290 (2012). 10.1002/mrm.24118 PMC3326213 22231619

[b46] Peng X. G. . Comparison of brown and white adipose tissue fat fractions in ob, seipin, and Fsp27 gene knockout mice by chemical shift-selective imaging and (1)H-MR spectroscopy. American journal of physiology. Endocrinology and metabolism 304, E160–167 (2013). 23149622 10.1152/ajpendo.00401.2012

[b47] Branca R. T. . Detection of brown adipose tissue and thermogenic activity in mice by hyperpolarized xenon MRI. Proceedings of the National Academy of Sciences of the United States of America 111, 18001–18006 (2014). 25453088 10.1073/pnas.1403697111 PMC4273360

[b48] Nakayama A., Bianco A. C., Zhang C. Y., Lowell B. B. & Frangioni J. V. Quantitation of brown adipose tissue perfusion in transgenic mice using near-infrared fluorescence imaging. Molecular imaging 2, 37–49 (2003). 12926236 10.1162/15353500200303103

[b49] Rice D. R., White A. G., Leevy W. M. & Smith B. D. Fluorescence Imaging of Interscapular Brown Adipose Tissue in Living Mice. Journal of materials chemistry. B, Materials for biology and medicine 3, 1979–1989 (2015). 10.1039/C4TB01914H PMC4442081 26015867

[b50] Azhdarinia A. . A peptide probe for targeted brown adipose tissue imaging. Nature communications 4, 2472 (2013). 10.1038/ncomms3472 PMC3806199 24045463

[b51] Ran C. . Design, synthesis, and testing of difluoroboron-derivatized curcumins as near-infrared probes for *in vivo* detection of amyloid-beta deposits. J. Am. Chem. Soc. 131, 15257–15261 (2009). 19807070 10.1021/ja9047043 PMC2784241

[b52] Ran C., Zhao W., Moir R. D. & Moore A. Non-conjugated small molecule FRET for differentiating monomers from higher molecular weight amyloid beta species. PloS one 6, e19362 (2011). 21559413 10.1371/journal.pone.0019362 PMC3084834

[b53] Zhang X. . Design and synthesis of curcumin analogues for *in vivo* fluorescence imaging and inhibiting copper-induced cross-linking of amyloid Beta species in Alzheimer’s disease. J. Am. Chem. Soc. 135, 16397–16409 (2013). 24116384 10.1021/ja405239v PMC3927838

[b54] Carpenter A. E. Image-based chemical screening. Nat. Chem. Biol. 3, 461–465 (2007). 17637778 10.1038/nchembio.2007.15

[b55] Lemieux G. A. . A whole-organism screen identifies new regulators of fat storage. Nat. Chem. Biol. 7, 206–213 (2011). 21390037 10.1038/nchembio.534 PMC3076723

[b56] Baker M. Academic screening goes high-throughput. Nat. Methods 7, 787–792 (2010).

[b57] Das B. C., McCartin K., Liu T. C., Peterson R. T. & Evans T. A forward chemical screen in zebrafish identifies a retinoic acid derivative with receptor specificity. PloS one 5, e10004 (2010). 20368991 10.1371/journal.pone.0010004 PMC2848850

[b58] Greenspan P., Mayer E. P. & Fowler S. D. Nile red: a selective fluorescent stain for intracellular lipid droplets. The Journal of cell biology 100, 965–973 (1985). 3972906 10.1083/jcb.100.3.965 PMC2113505

[b59] Bonilla E. & Prelle A. Application of nile blue and nile red, two fluorescent probes, for detection of lipid droplets in human skeletal muscle. The journal of histochemistry and cytochemistry: official journal of the Histochemistry Society 35, 619–621 (1987). 3559182 10.1177/35.5.3559182

[b60] Barneda D., Frontini A., Cinti S. & Christian M. Dynamic changes in lipid droplet-associated proteins in the “browning” of white adipose tissues. Biochimica et biophysica acta 1831, 924–933 (2013). 23376222 10.1016/j.bbalip.2013.01.015

[b61] Baboota R. K. . Capsaicin induces “brite” phenotype in differentiating 3T3-L1 preadipocytes. PloS one 9, e103093 (2014). 25072597 10.1371/journal.pone.0103093 PMC4114566

[b62] Asano H. . Induction of beige-like adipocytes in 3T3-L1 cells. The Journal of veterinary medical science/the Japanese Society of Veterinary Science 76, 57–64 (2014). 24065084 10.1292/jvms.13-0359 PMC3979956

[b63] Moreno-Navarrete J. M. . Irisin is expressed and produced by human muscle and adipose tissue in association with obesity and insulin resistance. The Journal of clinical endocrinology and metabolism 98, E769–778 (2013). 23436919 10.1210/jc.2012-2749

[b64] Weissleder R. A clearer vision for *in vivo* imaging. Nat. Biotechnol. 19, 316–317 (2001). 11283581 10.1038/86684

[b65] Alberts B., Johnson A. & Lewis J. Molecular Biology of the Cell, (Garland Science, New York, 2002).

[b66] Bassingthwaighte J. B. Overview of the processes of delivery: flow, transmembrane transport, reaction, and retention. Circulation 72, IV39–46 (1985). 2414031 PMC3646639

[b67] Papadopoulos S., Jurgens K. D. & Gros G. Protein diffusion in living skeletal muscle fibers: dependence on protein size, fiber type, and contraction. Biophys. J. 79, 2084–2094 (2000). 11023912 10.1016/S0006-3495(00)76456-3 PMC1301098

[b68] Beck R. & Schultz J. Hindrance of solute diffusion within membranes as measured with microporous membranes of known pore geometry. Biochim Biophys. Acta. 255, 273–303 (1972). 4334681 10.1016/0005-2736(72)90028-4

[b69] Haucke G., Czerney P., Ilge H.-D., Steen D. & Hartmann H. The effect of internal rotation on absorption and fluorescence of dye molecules. J. Mol. Struct. 411–416 (1990).

[b70] Kaplan M. A., Hays R. M. & Blumenfeld O. O. Membrane proteins and urea and acetamide transport in the human erythrocyte. The Journal of membrane biology 20, 181–190 (1975). 164554 10.1007/BF01870635

[b71] Demers A. . Identification of the growth hormone-releasing peptide binding site in CD36: a photoaffinity cross-linking study. The Biochemical journal 382, 417–424 (2004). 15176951 10.1042/BJ20040036 PMC1133797

[b72] Baranova I. N. . CD36 is a novel serum amyloid A (SAA) receptor mediating SAA binding and SAA-induced signaling in human and rodent cells. J Biol Chem 285, 8492–8506 (2010). 20075072 10.1074/jbc.M109.007526 PMC2832998

[b73] Coburn C. T., Hajri T., Ibrahimi A. & Abumrad N. A. Role of CD36 in membrane transport and utilization of long-chain fatty acids by different tissues. Journal of molecular neuroscience: MN 16, 117–121; discussion 151-117 (2001). 11478366 10.1385/JMN:16:2-3:117

[b74] Greenwalt D. E., Scheck S. H. & Rhinehart-Jones T. Heart CD36 expression is increased in murine models of diabetes and in mice fed a high fat diet. The Journal of clinical investigation 96, 1382–1388 (1995). 7544802 10.1172/JCI118173 PMC185760

[b75] Harmon C. M. & Abumrad N. A. Binding of sulfosuccinimidyl fatty acids to adipocyte membrane proteins: isolation and amino-terminal sequence of an 88-kD protein implicated in transport of long-chain fatty acids. The Journal of membrane biology 133, 43–49 (1993). 8320718 10.1007/BF00231876

[b76] Zhou D. . CD36 level and trafficking are determinants of lipolysis in adipocytes. FASEB journal: official publication of the Federation of American Societies for Experimental Biology 26, 4733–4742 (2012). 22815385 10.1096/fj.12-206862 PMC3475246

[b77] Williams K. J. & Fisher E. A. Globular warming: how fat gets to the furnace. Nat. Med. 17, 157–159 (2011). 21297605 10.1038/nm0211-157

[b78] Khalifeh-Soltani A. . Mfge8 promotes obesity by mediating the uptake of dietary fats and serum fatty acids. Nat. Med. 20, 175–183 (2014). 24441829 10.1038/nm.3450 PMC4273653

[b79] Sandoval A. . Fatty acid transport and activation and the expression patterns of genes involved in fatty acid trafficking. Archives of biochemistry and biophysics 477, 363–371 (2008). 18601897 10.1016/j.abb.2008.06.010

[b80] Jamal Z. & Saggerson E. D. Changes in brown-adipose-tissue mitochondrial processes in streptozotocin-diabetes. The Biochemical journal 252, 293–296 (1988). 3421907 10.1042/bj2520293 PMC1149137

[b81] Seydoux J. . Brown adipose tissue metabolism in streptozotocin-diabetic rats. Endocrinology 113, 604–610 (1983). 6223804 10.1210/endo-113-2-604

[b82] Burcelin R., Kande J., Ricquier D. & Girard J. Changes in uncoupling protein and GLUT4 glucose transporter expressions in interscapular brown adipose tissue of diabetic rats: relative roles of hyperglycaemia and hypoinsulinaemia. The Biochemical journal 291 (Pt 1), 109–113 (1993). 8471028 10.1042/bj2910109 PMC1132488

[b83] Ocloo A., Shabalina I. G., Nedergaard J. & Brand M. D. Cold-induced alterations of phospholipid fatty acyl composition in brown adipose tissue mitochondria are independent of uncoupling protein-1. *American journal of physiology*. Regulatory, integrative and comparative physiology 293, R1086–1093 (2007). 10.1152/ajpregu.00128.2007 17609311

[b84] Mirbolooki M. R., Constantinescu C. C., Pan M. L. & Mukherjee J. Quantitative assessment of brown adipose tissue metabolic activity and volume using 18F-FDG PET/CT and β3-adrenergic receptor activation. EJNMMI research 1, 30 (2011). 22214183 10.1186/2191-219X-1-30 PMC3250993

[b85] Kim H. . Effect of adipocyte beta3-adrenergic receptor activation on the type 2 diabetic MKR mice. American journal of physiology. Endocrinology and metabolism 290, E1227–1236 (2006). 16682489 10.1152/ajpendo.00344.2005

[b86] Xue Y. . FOXC2 controls Ang-2 expression and modulates angiogenesis, vascular patterning, remodeling, and functions in adipose tissue. Proceedings of the National Academy of Sciences of the United States of America 105, 10167–10172 (2008). 18621714 10.1073/pnas.0802486105 PMC2481379

[b87] Honek J. . Brown adipose tissue, thermogenesis, angiogenesis: pathophysiological aspects. Hormone molecular biology and clinical investigation 19, 5–11 (2014). 25390012 10.1515/hmbci-2014-0014

[b88] Peirce V., Carobbio S. & Vidal-Puig A. The different shades of fat. Nature 510, 76–83 (2014). 24899307 10.1038/nature13477

[b89] Ran C. & Moore A. Spectral Unmixing Imaging of Wavelength-Responsive Fluorescent Probes: An Application for the Real-Time Report of Amyloid Beta Species in Alzheimer’s Disease. Mol imag. biol. 14, 293–300 (2012). 10.1007/s11307-011-0501-7 PMC3229962 21739354

