# Supplementary material for: Curcumin analogues as selective fluorescence imaging probes for brown adipose tissue and monitoring browning
Source: Sci Rep. 2015 Aug 13;5:13116. doi: 10.1038/srep13116 (PMC4534785; doi:10.1038/srep13116)
Supplement: Supplementary Information [file srep13116-s1.pdf]

Curcumin analogues as fluorescence imaging probes for brown adipose tissue and monitoring browning

Xueli Zhang[1,2,3,6], Yanli Tian[1,4,6], Hongbin Zhang[5], Amol Kavishwar[1], Matthew Lynes[5], Anna-Liisa Brownell[1], Hongbin Sun[2], Yu-Hua Tseng[5], Anna Moore[1], and Chongzhao Ran[1]**

[1]Molecular Imaging Laboratory, Athinoula A. Martinos Center for Biomedical Imaging, Massachusetts General Hospital/Harvard Medical School, Boston, MA;

[2] School of Pharmacy, China Pharmaceutical University, Nanjing 210009, China;
[3]Department of pharmacy, ZhongDa Hospital, Southeast University;
[4]Department of Parasitology, Zhongshan School of Medicine, Sun Yat-Sen University, Guangzhou, China;
[5]Joslin Diabetes Center, Harvard Medical School, and Harvard Stem Cell Institute, Boston, MA 02215;
[6]These authors contributed equally to this work.

Corresponding authors: Chongzhao Ran, cran@nmr.mgh.harvard.edu
Anna Moore, amoore@helix.mgh.harvard.edu

Limited uptake mechanism studies with CRANAD-2:

A small molecule across cell-membrane can be mediated by simple diffusion and facilitated diffusion/transport. Fast simple diffusion often leads to non-specific uptake, and highly hydrophobic compounds prone to cross the cell membrane through the fast simple diffusion [1]. From our screening, most of the tested 38 dyes were highly hydrophobic. However, only CRANAD-2, -3, and Nile blue provided positive or negative contrasts, suggesting that the simple diffusion caused by high hydrophobicity was not the only factor in determining the BAT contrast, and facilitated diffusion/transport of CRANAD-2 and -3 into BAT was also very possible. To investigate the facilitated transporting of CRANAD-2, we compared the time courses of CRANAD-2 uptake in 3T3-L1 cells before and after differentiation. The uptake reached its maximal within 1 minute with undifferentiated 3T3-L1 cells (SI Fig.5a, left), indicating that the uptake is most likely due to simple diffusion. For differentiated 3T3-L1 cells, the uptake reached 62% of the maximal within 1 minute, and reached the plateau at 15 minute (SI Fig.5a, right), suggesting that partial accumulation of CRANAD-2 is likely due to facilitated diffusion mediated by receptors/transolcases expressed in differentiated 3T3-L1 cells, but not in preadipocytes. Nile blue, the negative contrast probe, rapidly reached the plateau in both differentiated and undifferentiated 3T3-L1 cells, suggesting that its uptake was primarily due to the fast simple diffusion (SI Fig.6a, b).

Through systemic comparison of gene expression in undifferentiated and differentiated 3T3-L1 cells, Sandoval et al found that CD36 was only present in differentiated cells but not in the preadipocytes [2]. CD36 is highly expressed on the surface of adipose cells and endothelial cells of capillaries in adipose tissue, but not on other large vessels and brain capillaries [3-6]. Recently, Bartelt et al reported that BAT regulates the metabolism of triglyceride through CD36 uptake [7,8]. Since CRANAD-2, like triglyceride, accumulates in oil droplets, we hypothesized that triglyceride and our curcumin analogues may share the same transporter/translocase. To test this hypothesis, we added both triglyceride and CRANAD-2 to the cell medium, and CRANAD-2 only was used as the control. A significant decrease in uptake was observed for the mixture of CRANAD-2 and triglyceride, evidenced by two-photon microscopy and quantitative imaging with IVIS imaging system (SI Fig.5b-d). However, no significant decrease in

uptake was observed for Nile Blue (SI Fig.5c and SI Fig.6c). These results indicated that triglyceride inhibited the uptake of CRANAD-2, probably due to binding competition for CD36. In addition, Wang et al showed that rosmarinic acid was a high affinity ligand for CD36 [9]. Very interestingly, the structure of rosmarinic acid is very similar to curcumin. These data suggested that curcumin and its analogues are possible ligands for CD36. However, more comprehensive mechanism studies are still needed.

Reference

1. Alberts, B., Johnson, A. & Lewis, J. *Molecular Biology of the Cell*, (Garland Science, New York, 2002).
2. Sandoval, A.*, et al.* Fatty acid transport and activation and the expression patterns of genes involved in fatty acid trafficking. *Archives of biochemistry and biophysics* **477**, 363-371 (2008).
3. Coburn, C.T., Hajri, T., Ibrahimi, A. & Abumrad, N.A. Role of CD36 in membrane transport and utilization of long-chain fatty acids by different tissues. *Journal of molecular neuroscience : MN* **16**, 117-121; discussion 151-117 (2001).
4. Greenwalt, D.E., Scheck, S.H. & Rhinehart-Jones, T. Heart CD36 expression is increased in murine models of diabetes and in mice fed a high fat diet. *The Journal of clinical investigation* **96**, 1382-1388 (1995).
5. Harmon, C.M. & Abumrad, N.A. Binding of sulfosuccinimidyl fatty acids to adipocyte membrane proteins: isolation and amino-terminal sequence of an 88-kD protein implicated in transport of long-chain fatty acids. *The Journal of membrane biology* **133**, 43-49 (1993).
6. Zhou, D.*, et al.* CD36 level and trafficking are determinants of lipolysis in adipocytes. *FASEB journal : official publication of the Federation of American Societies for Experimental Biology* **26**, 4733-4742 (2012).
7. Williams, K.J. & Fisher, E.A. Globular warming: how fat gets to the furnace. *Nat. Med.* **17**, 157-159 (2011).
8. Bartelt, A.*, et al.* Brown adipose tissue activity controls triglyceride clearance. *Nat. Med.* **17**, 200-205 (2011).
9. Wang, L.*, et al.* Discovery of antagonists for human scavenger receptor CD36 via an ELISA-like high-throughput screening assay. *J. Biomol. Screen.* **15**, 239-250 (2010).

Supplemental Figures

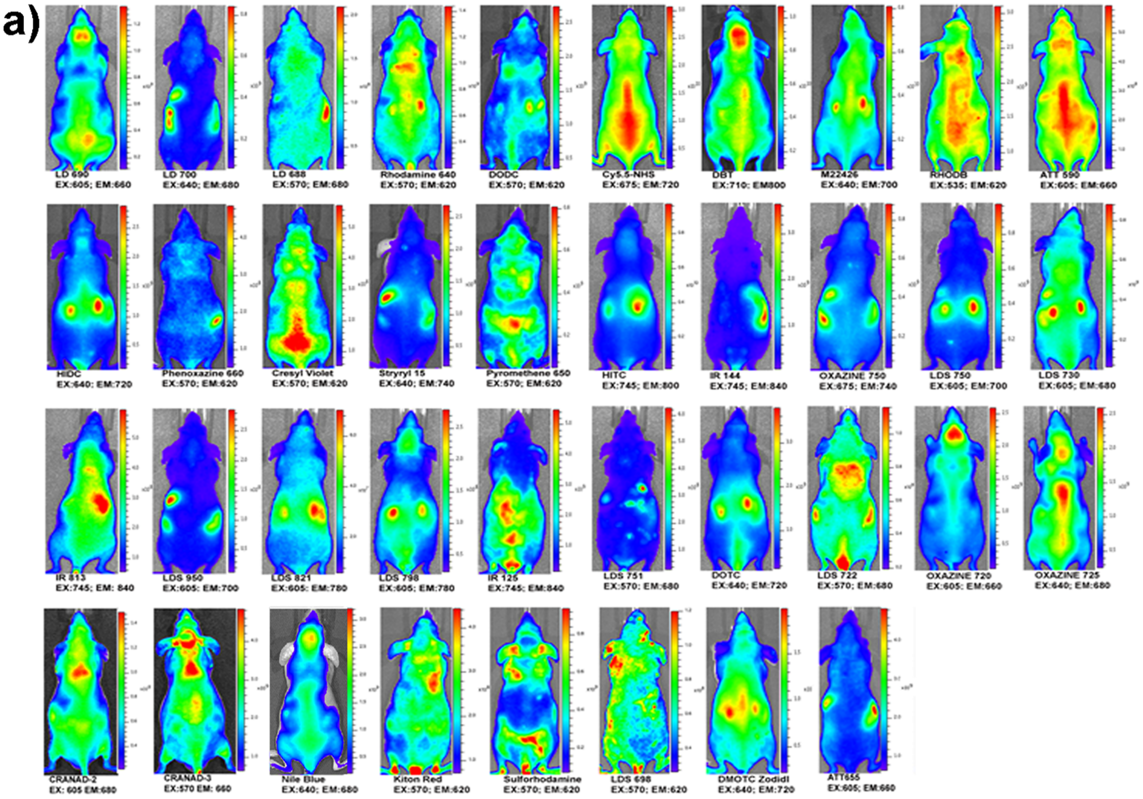

SI Figure 1 a) In vivo NIR images of mice injected with 38 fluorescent dyes. Corresponding excitation and emission wavelengths are listed for each image.

Dye list: 1.HITC, 2.IR144, 3.OX750, 4.LDS750, 5.LDS730, 6.LDS751, 7.DOTC, 8.LDS722, 9.OX720, 10.OX725, 11.LD690, 12.LD700, 13.LD688, 14.Rhodamine640, 15.DODC, 16.HIDC, 17.Phenox660, 18.Cresyl Violet, 19.KITON, 20.Sulforhodamine, 21.LDS698, 22.DMOTC, 23.Stryl15, 24.Pyromethene650, 25.Nile Blue, 26.Rhodamine, 27.B ATTO590, 28.ATT655, 29.DBT, 30.CRANAD-2, 31.CRANAD-3, 32.Cy5.5, 33.M2466 (Mitotracker deep red FM), 34 IR813, 35.LDS950, 36.LDS821, 37.LDS798, 38.IR125.

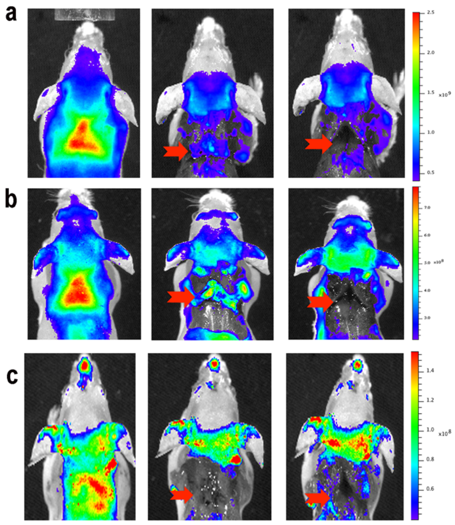

SI Figure 2 *Re-testing of the hits and validation of the signal with stepwise dissection and ex vivo histology.* (a-c) Re-testing with CRANAD-2 (a), CRANAD-3 (b), and LDS 722 (c). (left) Images of intact mice, (middle) images after skin and WAT were removed, and (right) images after BAT was dissected. In all cases, the signals were significantly decreased once the skin and WAT were dissected. CRANAD-2 and -3 showed higher signal before BAT removal than after BAT removal (a, b). LDS 722 had no apparent signal difference (c, middle), indicating that LDS 722 was not suitable for labeling BAT *in vivo*.

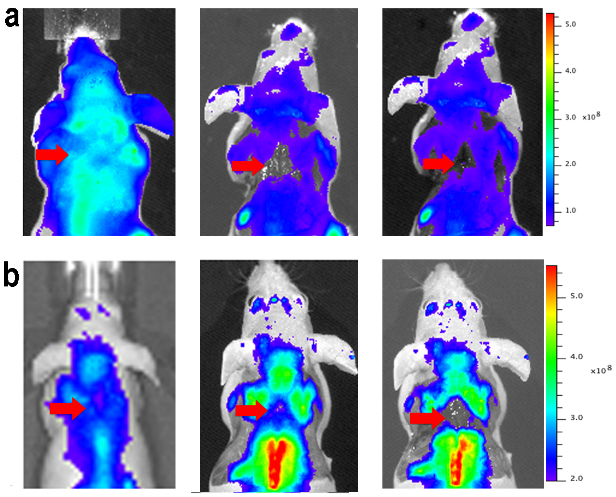

SI Figure 3 *In vivo re-testing of Rhodamine 640 and Phenoxazine 660 and validation of BAT signal using stepwise dissection procedure.* (a) Re-testing of Rhodamine 640 and stepwise dissection images. Image of the intact mouse (left); image after skin and WAT were removed (middle); image after BAT (red arrow) was removed (right). (b) Re-testing of Phenoxazine 660 and stepwise dissection images. Image of the intact mouse (left); image after skin and WAT were removed (middle); and image after BAT (red arrow) was removed (right). These images indicated that no significant signals were from BAT in both cases.

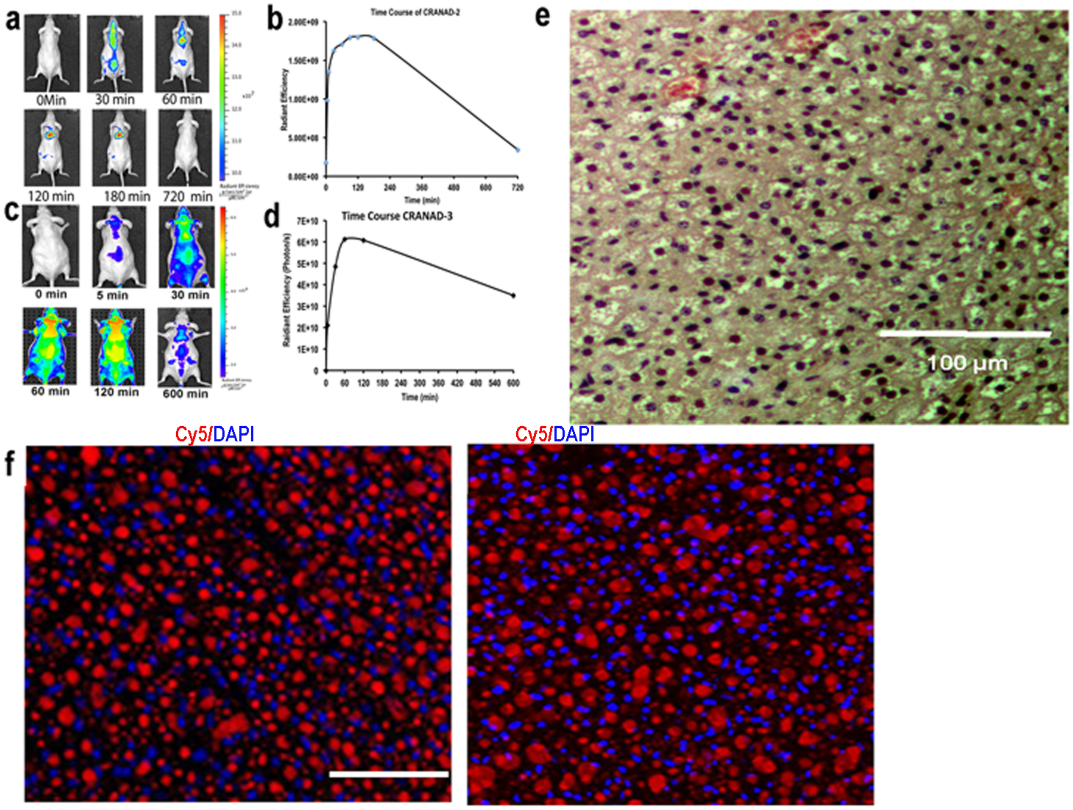

SI Figure 4 *Time course of CRANAD-2 and CRANAD-3, ex vivo histological staining.* (a) Images of animals injected intravenously with CRANAD-2 at different time points. (b) Quantitative analysis of interscapular BAT images of animals injected with CRANAD-2 at different time points. (c) Images of animals injected with CRANAD-3 at different time points after intravenous injection. (d) Quantitative analysis of interscapular BAT images of animals injected with CRANAD-3 at different time points. (e) H&E staining for *ex vivo* BAT tissue. Oil droplets were white, and nuclei were purple/blue (10x). (f) *Ex vivo* histology of animals injected with CRANAD-2 (left) and CRANAD-3 (right). Oil droplets were stained by CRANAD-2, or -3 (red), and nuclei were labeled with DAPI (blue) (10x). Scale bar: 100 micron.

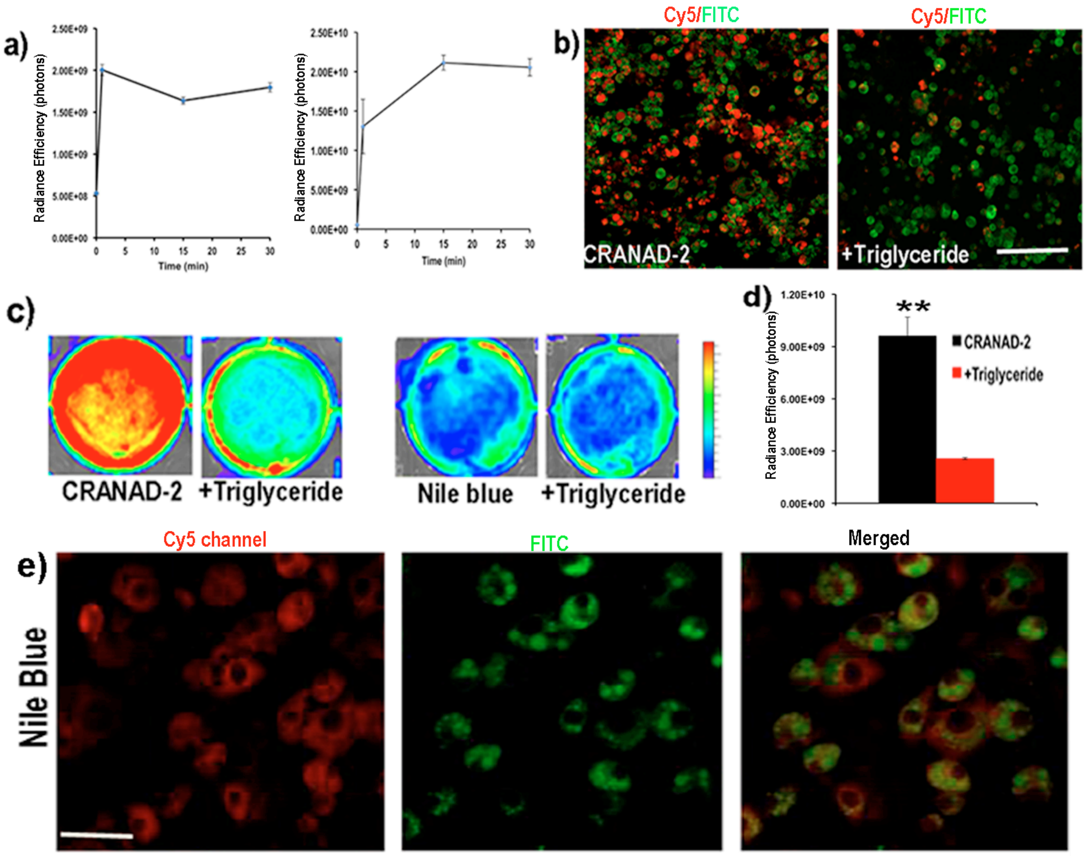

SI Figure 5 *Limited uptake mechanism studies*. (a) The uptake time course of CRANAD-2 in undifferentiated (left) and differentiated 3T3-L1 cells. The fast uptake phase indicates simple diffusion. (b) Two-photon microscopic images of CRANAD-2 alone (left), with triglyceride competition (right). Red - CRANAD-2 signal, and green – autofluorescence of the cells. Scale bar: 100 micron. Apparent loss of CRANAD-2 accumulation in oil droplets was observed in triglyceride- treated cells. (c) Representative quantitative images of the cells treated with triglyceride obtained with IVIS imaging system for CRANAD-2 and Nile blue. (d) Quantitative analysis of the images of CRANAD-2 (n=3) in (c). (e) Two-photon cell imaging of 3T3-L1 adipose cells with Nile blue (left: Nile blue in the cytoplasm, middle: Nile blue in oil droplets, right: merged). Scale bar: 50 micron.

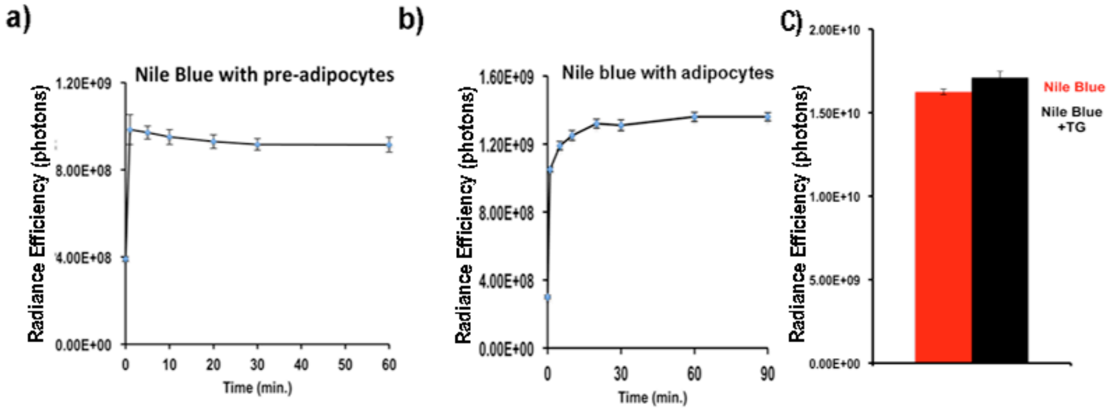

SI Figure 6 The uptake time course of Nile blue with undifferentiated (a) and differentiated 3T3-L1 cells (b), (c) the uptake of Nile blue in differentiated 3T3-L1 cells without (red) and with triglyceride (black).

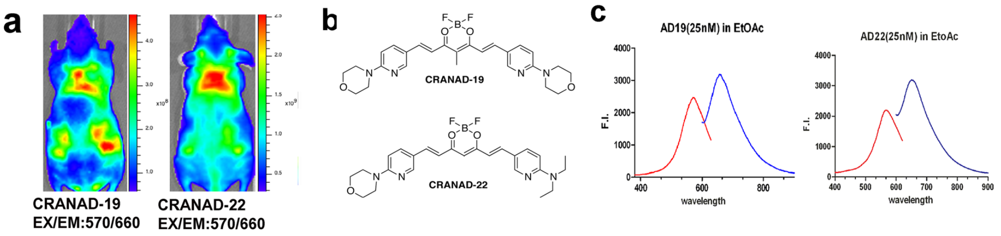

SI Figure 7 *Lead optimization based on CRANAD-3.* (a) NIR images CRANAD-19 and -22. (b) Chemical structures of CRANAD-19 and -22. (c) Excitation (red line) and emission spectra (blue line) of CRANAD-19 and -22.

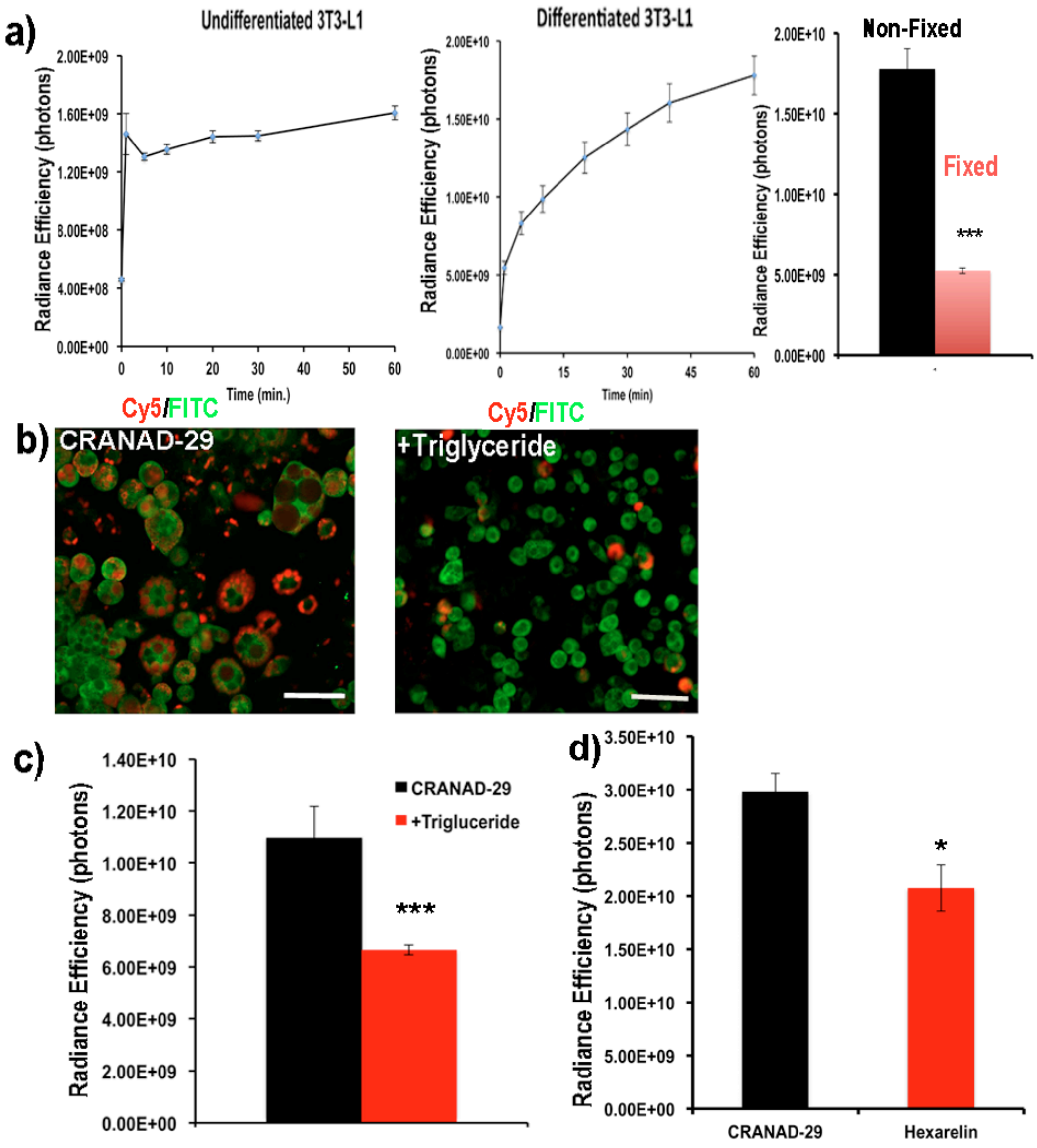

SI Figure 8 *Limited uptake mechanism studies for CRANAD-29.* (a) The uptake time course of CRANAD-29 in undifferentiated (left) and differentiated 3T3-L1 cells (middle), and the uptake blocking with glutaraldehyde fixed 3T3-L1 cells (right). (b) Two-photon microscopic images of CRANAD-29 alone (left), with triglyceride competition (right). Red - CRANAD-29 signal, and green – autofluorescence of the cells. Scale bar: 100 micron. Apparent loss of CRANAD-29 accumulation in oil droplets was observed in cells treated with triglyceride. (c) Quantitative analysis images of the cells treated with triglyceride obtained with IVIS imaging system (n = 3). (d) Quantitative analysis images of the cells treated with CD36 specific ligand Hexarelin obtained with IVIS imaging system.

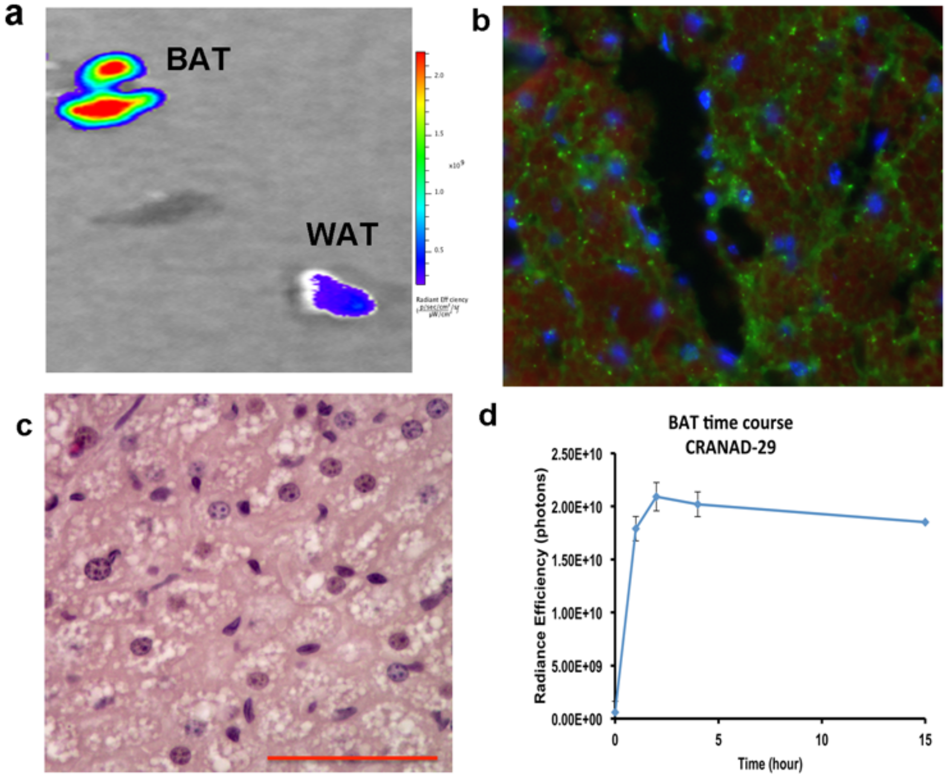

SI Figure 9 (a) NIR imaging of *ex vivo* BAT and WAT at 4 hours after CRANAD-29 injection. (b-c) Representative fluorescence microscopic image (b) and H&E staining image of *ex vivo* BAT tissue slice of the animals injected with CRANAD-29. In (b), red-oil droplets, and green-autofluorescence of cells, and blue-DAPI. Scale bar: 150 micron. (d) The time course of CRANAD-29 fluorescence signal after intravenous injection.

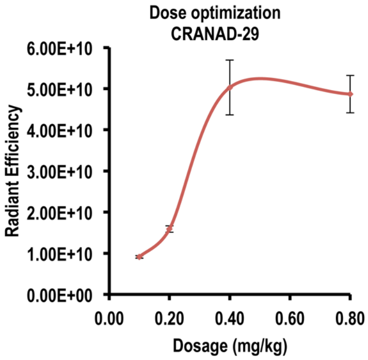

SI Figure 10 Dose optimization for *in vivo* studies with CRANAD-29.

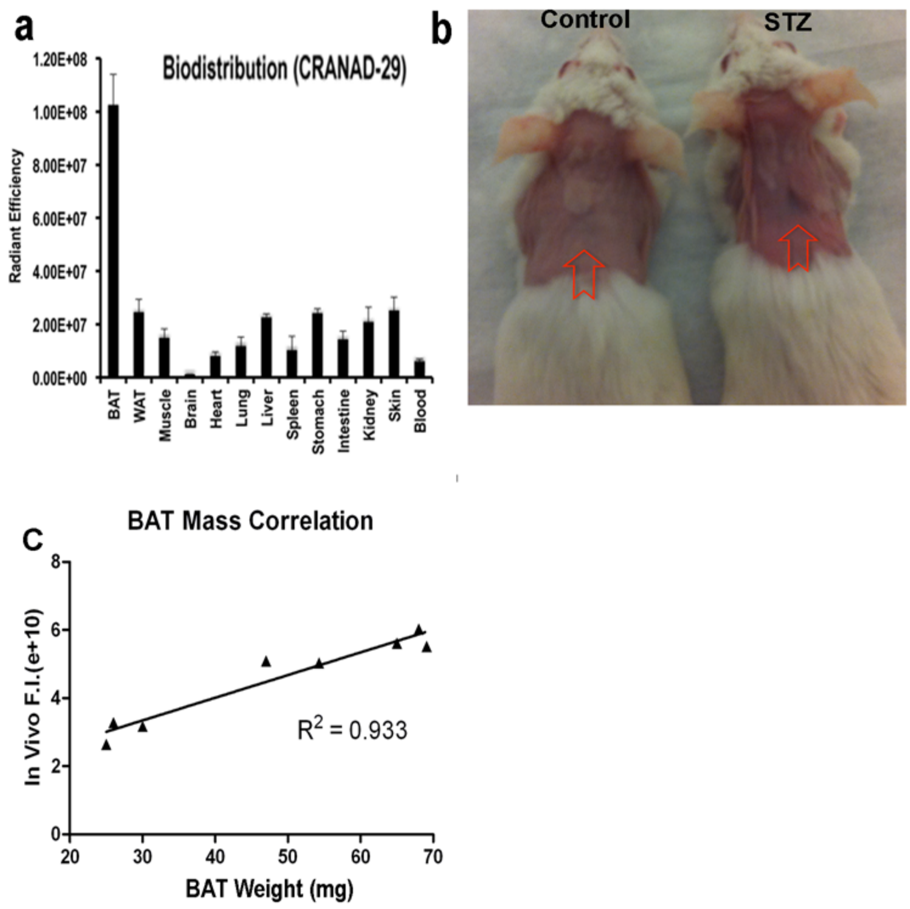

SI Figure 11 (a) Bio-distribution of CRANAD-29 at 4 hours post intravenous injection of CRANAD-29. Our data indicated that the highest CRANAD-29 uptake was in BAT. (b) Representative photographic images of a control mouse (left) and an STZ-treated mouse (right). Red arrows point to interscapular BAT. (c) Correlation between NIR fluorescence signal and the weight of the dissected BAT.

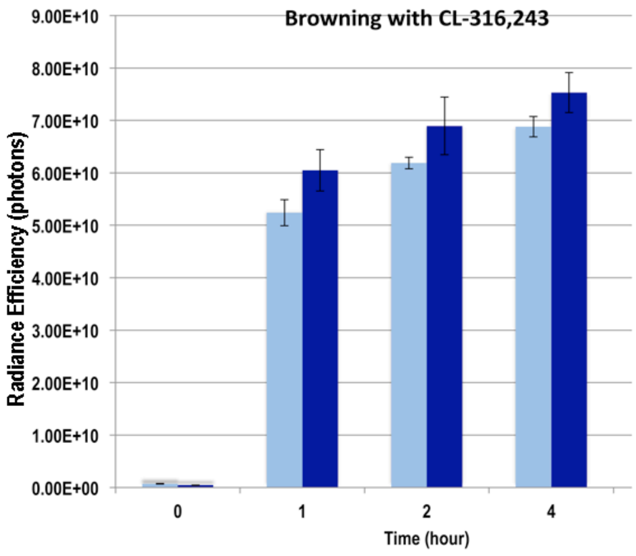

SI Figure 12 Quantitative analysis of the fluorescent signals of interscapular BAT at 1, 2, and 4 hours post CRANAD-29 injection for the CL 316,243 treated group (deep blue) and the control group (light blue).
